# Identification of antimicrobial compounds in *Dipsacus inermis* via phytochemical profiling, *in vitro* assessment, and advanced computational techniques

**DOI:** 10.1371/journal.pone.0341424

**Published:** 2026-02-06

**Authors:** Drakhshaan, Tahir Ali Chohan, Aisha Qayyum, Abdullah R. Alzahrani, Ahd A. Mansour, Hayat Ali Alzahrani, Abida Khan, Muhammad Umer Khan

**Affiliations:** 1 Institute of Pharmaceutical Sciences, University of Veterinary and Animal Sciences Lahore, Lahore, Pakistan; 2 Department of pediatric medicine, Fatima memorial Hospital, Lahore, Pakistan; 3 Department of Pharmacology and Toxicology, Faculty of Medicine, Umm Al-Qura University, Al-Abidiyah, Makkah, Saudi Arabia; 4 Medical Laboratory Sciences Department, Fakeeh College for Medical Sciences, Jeddah, Saudi Arabia; 5 Department of Medical Laboratory Technology, College of Applied Medical Sciences, Northern Border University, Arar, Saudi Arabia; 6 Center for Health Research, Northern Border University, Arar, Saudi Arabia; 7 Institute of Molecular Biology and Biotechnology, University of Lahore, Lahore, Pakistan; University of Nairobi Faculty of Health Sciences, KENYA

## Abstract

Antimicrobial resistance (AMR) poses a major challenge in treating infections such as pneumonia and typhoid fever, necessitating novel therapeutics. Plant-derived natural products provide a promising alternative. This study evaluated dichloromethane (DCM) and methanol (MeOH) extracts of *Dipsacus inermis* against six bacterial strains: *Staphylococcus aureus*, *Bacillus subtilis*, *Escherichia coli*, *Pseudomonas aeruginosa*, *Salmonella typhi*, and *Enterobacter aerogenes*. Antibacterial activity was assessed following standardized CLSI guidelines for both zone of inhibition (ZOI) and minimum inhibitory concentration (MIC) assays. The DCM extract demonstrated superior activity, with ZOI values of 17.87 ± 0.23 mm (*S. aureus* and *E. aerogenes*) and 16.83 ± 0.29 mm (*S. typhi*), and MICs of 1.562 mg/mL (*B. subtilis* and *E. aerogenes*) and 12.5 mg/mL (*S. aureus*, *S. typhi*, *E. coli*, and *P. aeruginosa*). One-way ANOVA followed by pairwise post-hoc comparisons confirmed significant differences among extract concentrations and relative to the reference control, highlighting dose-dependent potency. GC-MS and HPLC analyses identified multiple bioactive compounds, primarily terpenoids and steroids. All identified compounds were subjected to in silico studies against DNA gyrase B, tyrosyl-tRNA synthetase, PBP2X, PBP4, and DHFR. Compounds DI10 and DI31 emerged as potent multi-target leads, while DI22 exhibited selective activity against PBP4. ADMET profiling indicated favorable pharmacokinetics, high intestinal absorption, and minimal toxicity risks. DFT and MESP analyses revealed electronic features and reactive sites critical for ligand-protein interactions. Molecular dynamics simulations confirmed stable protein-ligand complexes, with RMSD stabilizing at 1.5-2.5 Å, compact conformations (Rg: 16.3-21.6 Å), persistent hydrogen bonds, and favorable binding free energies (−45 to −52 kcal/mol) via MM-PBSA. These integrated *in vitro* and *in silico* findings indicate that DCM-derived compounds, particularly DI10 and DI31, are primarily responsible for the observed antibacterial activity and represent promising candidates for antimicrobial drug development.

## Introduction

Natural compounds derived from different sources, including plants, animals, and microorganisms, have historically been an invaluable foundation for drug discovery [1,2]. In particular, components isolated and purified from plants are esteemed for their potential as precursors in the development of treatments for diverse health conditions [3]. Numerous approved therapeutics originate from natural products. The widespread availability of these resources significantly contributes to the identification and development of novel therapeutic compounds. This process involves the extraction and characterization of bioactive molecules, which are crucial for the synthesis of new medicinal products [4]. Historical records indicate that the use of plants in regions such as India, China, and Egypt extend to approximately 5000 years ago, while in Central Asia, this practice is traced back to about 2500 years ago. This long-standing tradition underscores the integral role of plant-based therapies in the evolution of human healthcare systems across various cultures [5].

Antimicrobial resistance (AMR) represents a critical global public health challenge, as it leads to ineffective conventional treatments and complicates the management of infections caused by resistant strains, such as *Staphylococcus aureus* (causing skin infections and pneumonia), *Escherichia coli* (associated with urinary tract infections), *Pseudomonas aeruginosa* (linked to respiratory infections in immunocompromised individuals), *Bacillus subtilis* (which can cause gastrointestinal disturbances), *Salmonella typhi* (responsible for typhoid fever), and *Enterobacter aerogenes* (associated with nosocomial infections). The growing issue of AMR underscores the urgent need for innovative strategies to combat resistant strains and sustain the efficacy of existing therapeutic agents [6,7]. A growing number of reports from around the globe have attested to the antimicrobial properties of medicinal herbs. According to the WHO, 80% of people on the planet utilize plant extracts or their active ingredients in traditional medicine. Plant-based antibiotics are gaining traction as alternatives to traditional antimicrobials. As research and clinical trials progress, it is crucial to acknowledge that their use in clinical practice remains limited, with most studies still in the early stages [8]. Further studies underscore the potential of plant-derived substances to develop new therapeutic agents against microbial infections [9,10].

*Dipsacus Inermis (D. inermis*) is commonly referred to as the Chinese teasel or Himalayan Teasel. *D. inermis* is a perennial plant belonging to the *Caprifoliaceae* family, genus *Dipsacus*, comprising approximately 20 species. It is native to China, Europe, and tropical Africa, and has since become widespread across East Asia and other regions globally [11]. The plant is easily identifiable by its solitary, circular flower heads borne on long, leafless, channeled stalks, with flowering occurring from June to September. Traditionally, *D. inermis* has been valued in Chinese medicine for its diverse therapeutic properties [12] and has historically been used to manage ailments such as Lyme disease, fibromyalgia, and bacterial infections [13]. Previous studies have demonstrated that various *Dipsacus* species, particularly *D. inermis*, possess significant antibacterial and antifungal activity as evidenced by disc-diffusion (zone-of-inhibition) assays, providing empirical evidence of their antimicrobial potential against clinically relevant bacterial strains [14,15]. *Dipsacus* is well known for producing triterpenoid compounds. 34 triterpenoid compounds, numbered, have been identified from *D. inermis, D. azureus, and D. laciniatus* [16].

This study was aimed to investigate the antibacterial potential of dichloromethane (DCM) and methanol (MeOH) extracts of *Dipsacus inermis* against six bacterial strains: *S. aureus*, *E. coli*, *P. aeruginosa*, *B. subtilis*, *S. typhi*, and *E. aerogenes*. The extracts were screened using GC-MS and HPLC to identify the active components responsible for the antibacterial activity. Additionally, *in silico* docking studies of the identified compounds were conducted against bacterial proteins, including DNA gyrase B, tyrosyl-tRNA-synthetase, PBP2X, PBP4, and dihydrofolate reductase (DHFR), to determine the lead compound responsible for the antibacterial potential of *D. inermis*. This study aims to contribute to future drug discovery efforts involving lead compounds.

## Materials and methods

### Plant collection and extraction

The whole plant of *D. inermis* was collected in August 2023 from Muzaffarabad, Kashmir, Pakistan. The plant specimen was assigned voucher number UEH-P40021 and identified by Dr. Ghulam Yaseen, an expert in plant taxonomy, currently serving as assistant professor in the Department of Botany, Division of Science & Technology, University of Education, Lahore, Pakistan. The whole plant was washed, let to dry naturally in the shade, and then processed in an electric grinder to create a coarse powder [17]. *D. inermis* whole plant was subjected to maceration using solvents MeOH and DCM at room temperature with frequent agitation. Subsequently, the extracts were concentrated to a dry state using a rotary evaporator at 40 ℃. The extracts from each solvent were weighed, prepared stock solution in DMSO (100 mg/mL) and kept at 4.0 ℃ for further analysis [18].

### Antibacterial activity

#### Test bacterial strains.

The antibacterial activity of *D. inermis* extracts was evaluated against both Gram-positive (*Staphylococcus aureus* ATCC 29213, *Bacillus subtilis* ATCC 6633) and Gram-negative (*Escherichia coli* ATCC 25922, *Pseudomonas aeruginosa* ATCC 27853, *Salmonella typhi* ATCC 14028, *Enterobacter aerogenes* ATCC 13048) bacterial strains. All strains were pre-cultured overnight at 37°C in Mueller Hinton broth (MHB) using a rotary shaker. Bacterial suspensions were standardized to approximately 10^8 cells/mL according to the 0.5 McFarland standard. For experimental use, inocula were further diluted in agar medium to achieve a final concentration of 10^5 CFU/mL. The pH of the medium was adjusted to 7.0 to ensure optimal bacterial growth and assay reproducibility.

#### Agar well diffusion method (Zone of inhibition, ZOI).

The antibacterial activity of MeOH and DCM extracts of *D. inermis* was measured using a slightly modified disc diffusion method reported previously [19]. Mueller Hinton Agar (MHA) was autoclaved, poured into sterilized Petri dishes, and allowed to solidify under aseptic conditions, with a total volume of 25 mL per plate. Wells of 6.0 mm diameter were created using a sterile gel borer. Gram-positive (*S. aureus*, *B. subtilis*) and Gram-negative (*E. coli*, *P. aeruginosa*, *S. typhi*, *E. aerogenes*) bacterial strains were pre-cultured overnight at 37°C in MHB using a rotary shaker to ensure active growth. For the assay, 10 mL of sterile agar medium in each Petri dish was overlaid with 15 mL of agar seeded with the bacterial suspension, resulting in a final inoculum concentration of 10^5 CFU/mL. The pH of the medium was adjusted to 7.0 using a calibrated pH meter to ensure optimal growth conditions. Subsequently, 50 µL of each extract at concentrations of 100 and 50 µg/mL was added to the respective wells. Gentamicin (10 µg/disk) was used as a positive control, while 1.0% DMSO (v/v) served as a negative control, which did not inhibit bacterial growth. Plates were kept at 4°C for 30 minutes to allow uniform diffusion of the compounds into the agar and then incubated at 37°C for 24 hours. After incubation, the diameter of the inhibition zones around the wells was measured in millimeters using a caliper [20]. Each test was performed in triplicate independent experiments, performed on different days, which strengthens reproducibility, and zone diameters are presented as mean ± SD (n = 3). The activity of extracts was categorized as sensitive (S), intermediate (I), or resistant (R) according to Gentamicin CLSI breakpoints, as indicated for Enterobacterales (S ≥ 18 mm; I 15–17 mm; R ≤ 14 mm) [21]. For *P. aeruginosa* the gentamicin disk-diffusion interpretive categories were removed in later CLSI editions and thus are not applied; for *S. aureus* and *B. subtilis* CLSI does not provide disk-diffusion breakpoints for gentamicin, therefore raw zone diameters are reported without categorical classification.

### Statistical analysis.

All experiments were conducted in triplicate, with each assay independently repeated three times. Results are presented as mean ± SD to ensure accuracy and reproducibility. The antibacterial activities of the MeOH and DCM extracts at concentrations of 50 and 100 µg/mL against each bacterial strain were compared with Gentamicin (10 µg) using one-way analysis of variance (ANOVA) in Microsoft Excel (2021/365). Following ANOVA, pairwise post-hoc comparisons were performed to determine specific differences between extract types and concentrations: DCM 100 µg/mL vs MeOH 100 µg/mL (@), DCM 50 µg/mL vs MeOH 50 µg/mL (#), DCM 100 µg/mL vs DCM 50 µg/mL ($), and MeOH 100 µg/mL vs MeOH 50 µg/mL (&). Statistical significance is indicated by the corresponding symbols, with one, two, or three symbols representing p < 0.05, p < 0.01, and p < 0.001, respectively.

#### Broth microdilution method (minimum inhibitory concentration, MIC).

The MIC of MeOH and DCM extracts of *D. inermis* was determined using a standard broth microdilution method with slight modifications [22–26]. Sterile 96-well microliters plates were used for the assay. Wells A1–A12 were designated as the positive control, containing 170 µL of Mueller Hinton broth (MHB), 20 µL of vancomycin, and 10 µL of bacterial culture. Wells B1–B12 served as the negative control, containing 190 µL of MHB, 10 µL of bacterial inoculum, and 1.0% DMSO (v/v). The positive control demonstrated effective bacterial inhibition, whereas the negative control showed no inhibition, confirming assay validity. The remaining wells from rows C to H were initially filled with 100 µL of MHB. MeOH extract of *D. inermis* was added at concentrations of 50, 25, 12, 6.25, 3.125, and 1.562 mg/mL to wells C1–C6, while DCM extract at the same concentrations was added to wells C7–C12. Two-fold serial dilutions were performed by transferring 100 µL from each well downward across the rows, with 100 µL discarded from the last wells in row H, ensuring consistent final volumes. This procedure resulted in a final series of decreasing concentrations for each extract in the assay wells. Following the addition of extracts, 10 µL of bacterial suspensions (prepared as described in the Test Bacterial Strains section) was added to each well. Plates were incubated at 37°C for 24 hours to allow bacterial growth. After incubation, 20 µL of 0.01% resazurin solution was added to each well, and plates were further incubated at 37°C for 2–4 hours to allow sufficient reduction of resazurin by metabolically active bacteria. The color change from blue to pink was monitored: blue indicated inhibition of bacterial growth, whereas pink indicated viable, metabolically active bacteria due to the reduction of resazurin to resorufin. All experiments were performed in triplicate, with results expressed as mean ± SD, ensuring reliability and reproducibility of the assay. MIC values for *S. aureus* and *B. subtilis* were interpreted according to CLSI guidelines, whereas CLSI does not provide vancomycin susceptibility breakpoints for *E. coli*, *P. aeruginosa*, *S. typhi*, and *E. aerogenes*; therefore, MIC values for these strains are reported without categorization [21].

### GC-MS analysis

Identification of bioactive compounds present in DCM extract of *D. inermis* was performed by GC-MS [14,27–33]. The dried DCM extract was dissolved in dichloromethane and filtered through a 0.22 µm syringe filter before injection. A volume of 1.0 µL of the prepared sample was injected with a split ratio of 20:1. The injector temperature was maintained at 280 °C. Helium (He, 99.9%) was used as the carrier gas at a constant flow rate of 1.0 mL/min [34]. The GC oven was initially set to 50 °C and held for 3.0 min, followed by a temperature increase at 10 °C/min until reaching 280 °C. Chromatograms were recorded in full scan mode throughout the analysis. Compound identification was conducted by comparing retention times and mass spectra with the NIST Replib and MAINLIB spectral libraries, along with observed fragmentation patterns. This approach ensures reliable identification, and several detected compounds were consistent with those previously reported in *Dipsacus* species [35].

### HPLC-UV/Vis analysis

HPLC analysis was carried out using a Waters liquid chromatography system, comprising a Model 600 solvent delivery pump and a 2996 photodiode array detector, operated via Empower version 2 software. Compound separation was performed on a Prodigy ODS (3) C18 reversed-phase column (4.6 × 150 mm, 5.0 μm particle size), maintained at 30 ± 1 °C in a Jetstream2 Plus column oven. Data were collected across a wavelength range of 200–500 nm, with quantitative determinations conducted at the specific maximum absorbance wavelengths of the analytes. A 20 μL volume of each sample was injected. The mobile phase consisted of a mixture of water and acetonitrile (93:7, v/v) containing 3.0% acetic acid as the starting composition for a gradient elution, and online degassing was performed using a Biotech DEGASi Compact degasser. Gradient elution was carried out following previously published protocols [36,37]. Sample solutions were obtained by centrifugation, and the resulting supernatants were used for injection into the HPLC system. Stock solutions of phenolic standards (berberine, ferulic acid, eugenol, reserpine, quercetin, caffeic acid, rutin, and chlorogenic acid) were prepared at a concentration of 1.0 mg/mL in 10 mL of methanol. To create calibration curves, these stock solutions were diluted with the mobile phase to produce a series of mixed standard solutions with concentrations ranging from 10 to 200 μg/mL and 0.25 to 20 μg/mL, which were then analyzed via HPLC-UV/Vis. Solid extracts were dissolved in the mobile phase at a ratio of 1:1 (w/v), with the resulting concentrations (μg/mL) directly reflecting the total content (μg/mg) in the extract. Following dissolution, the mixtures were centrifuged at 12,000 × g and filtered prior to chromatographic analysis. Identification and quantification of individual phenolic compounds were carried out using standard references and expressed as micrograms per gram (μg/g) of the dried extract [38,39]. Calibration curves were prepared using standard solutions across relevant concentration ranges, ensuring accurate quantification, and the use of reference standards along with precise chromatographic conditions provides high confidence in compound identification and quantification [40,41].

### *In-silico* studies

#### Docking validation, structure preparation and molecular docking studies.

Molecular docking has been used to determine the binding orientation and affinity between a ligand and a biological macromolecule (often a protein) within the contexts of structural biology and drug discovery [42–44]. This technique aimed to find potential binding sites and evaluate the feasibility of ligand-protein interactions by simulating the intermolecular interactions and energetics that govern the formation of stable complexes between the ligand and the target biomolecule [45]. The co-crystal structures of five bacterial proteins were obtained from the RCSB PDB database [46], selected for their essential roles in bacterial cell wall synthesis and protein production as reported in previous studies [47,48]. The selected bacterial targets DNA gyrase B, tyrosyl-tRNA synthetase, PBP2X, PBP4, and DHFR are critical for DNA replication, protein synthesis, cell-wall biosynthesis, and folate metabolism, respectively. DNA gyrase B and tyrosyl-tRNA synthetase are universally present in all six bacterial strains tested in this study (*S. aureus, B. subtilis, E. coli, P. aeruginosa, S. typhi, and E. aerogenes*) [49], while PBP2X and PBP4 are particularly relevant for Gram-positive strains (*S. aureus* and *B. subtilis*) [50], and DHFR is essential in all strains for nucleotide and amino-acid biosynthesis [51]. These proteins have also been widely reported in previous computational studies as validated antibacterial targets, supporting their selection for docking with the identified plant compounds [52–56]. These proteins included *S. aureus* tyrosyl-tRNA synthetase (PDB ID: 1JIJ) [57], DNA gyrase B (PDB ID: 4DUH) [58], PBP2X from *S. pneumoniae* (PDB ID: 5OJ0) [59], PBP4 from *S. aureus* (PDB ID: 1TVF) [60] and DHFR from *S. aureus* (PDB ID: 2W9S) [61]. To validate the docking method, the co-crystalized ligands 4,5’-bithiazole, SB-239629, cefepime, and trimethoprim with their respective structures (4DUH, 1JIJ, 5OJ0, and 2W9S, respectively) were redocked again using Maestro software. Before docking, protein structures were rigorously prepared by removing solvent molecules, correcting missing atoms, and performing geometric optimization. These steps are essential to ensure the structural integrity and reliability of protein models [62].

The 3D structures of these proteins were prepared in Maestro using the default settings. To determine the phytoconstituent responsible for the DNA Gyrase B, tyrosyl-tRNA-synthetase, PBP2X, PBP4, and DHFR inhibitory potential of *D. inermis*, molecular docking experiments were conducted*.* Consequently, drug-like compounds identified by GC-MS analysis from the DCM extract and HPLC-PDA analysis from the MeOH extract of *D. inermis* [63–66] were docked into 1JIJ, 4DUH, 5OJ0, and 1TVF to reveal their binding affinity. A grid was created around the co-crystallized ligand using the Glide grid module to predict the *S. aureus* (PDB ID: 1TVF) protein, defining the search space for docking. This process involves generating receptor grids based on the binding sites of the ligand. The Glide tool within Maestro [67] was used to dock compounds into the binding site of DNA Gyrase B, tyrosyl-tRNA synthetase, PBP2X, PBP4, and DHFR. The docking employed the Glide XP mode, a technique for evaluating docking performance. After docking, the results were analyzed using the Maestro interface, allowing visualization of the obtained poses [68–70].

#### Structural interaction fingerprint (SIFt) analysis.

The fingerprint encapsulates the interaction pattern of the complex, offering a means to organize and analyse the complex [71]. The SIFt panel within Maestro was employed to generate interaction fingerprints for docking complexes of inhibitors with 1JIJ, 4DUH, 5OJ0, 1TVF, and 2W9S. The input files included the receptor grid and ligands. Once the fingerprint was created, the results were visualized in an Excel sheet, highlighting the residues and interactions that significantly contributed to the binding. Different colors were given to indicate the type of interaction exhibited by the residues, with 1.0 and 0.0 denoting the presence and absence of interaction, respectively [72].

#### ADMET studies.

ADMET stands for Absorption, Distribution, Metabolism, Excretion, and Toxicity. For all tentatively identified compounds, key properties such as intestinal absorption, distribution, metabolism, and excretion were analyzed using the SWISS ADMET web server [73]. The PkCSM-pharmacokinetics web tool, which is freely accessible, is an innovative approach for predicting and optimizing small-molecule ADMET properties. It utilizes graph-based signatures and experimental data for its prediction [74].

#### DFT studies/MESP/HOMO/LUMO analysis.

In line with established methodologies as referenced in the literature [75], DFT calculations were rigorously conducted using the Gaussian 09 software set to its default settings. All computational calculations were consistently performed using the B3LYP functional in combination with the SVP basis set, providing a dependable assessment of the electronic structures of the atoms and molecules involved. The primary objective was to determine critical molecular parameters such as optimized geometries, frontier molecular orbital (FMO) energies, reactivity descriptors, and molecular electrostatic potential (MEP) maps. These descriptors are fundamental for interpreting the chemical behavior and reactivity profiles of the compounds under investigation, offering valuable implications for both theoretical modeling and practical applications. Checkpoint files generated during these simulations were further analyzed using GaussView 6.0 to explore electron density distributions and potential energy surfaces in greater detail.

#### Molecular dynamic (MD) simulations.

The identified compounds DI31 and DI22 were subjected to MD simulations to enhance the stability of their chemical structures bound to proteins (DNA Gyrase B, tyrosyl-tRNA synthetase, and PBP4, respectively). This involved a series of steps using software Amber 20: assigning partial atomic charges to ligands with the antechamber module, integrating missing hydrogens, and preparing the system for simulation with the Leap module. Force fields-ff14SB for the protein and generalized Amber force field (GAFF) for the ligand were employed [76]. The process included neutralizing the proton-containing protein, solvating the complex, and saving the solvated complex in PDB format, along with preparing parameter and coordinate files. Stepwise minimization was carried out to eliminate any structural clashes within the system, focusing on the protein, ligand, and the entire complex in successive phases. Each minimization stage involved specific steps and adjustments to ions and solvent systems. The system underwent heating and equilibration stages to stabilize it at 300 K, followed by MD production for 100 ns under specific temperature and pressure conditions. Evaluations were performed using various parameters, such as RMSD, RMSF, SASA and Radius of Gyration (Rg) using the Amber 20 CPPTRAJ module. RMSD values were determined for ligands, protein pockets, and apoproteins over the 100 ns simulation time, providing insights into the system’s behavior and stability.

#### Binding free energy calculations by MMPBSA/MMGBSA.

The study estimated binding free energies (BFE) by analyzing snapshots from molecular dynamics (MD) simulations. To compute these energies, the molecular mechanics MMPBSA/MMGBSA modules within the AMBER 20 software were utilized [77]. The data was derived from the final 10 nanoseconds of 1000 snapshots taken from three distinct complex systems: DNA gyrase B-DI31, tyrosyl-tRNA synthetase-DI31 and PBP4-DI22. These calculations aimed to discern the energy difference between the inhibitor complex system (ΔG_com_) and the free protein (ΔG_ache_) according to equation (1).


$$\Delta {\text{G}}_{\text{bind}}=\Delta \text{H}-\text{T}\Delta \text{S}=\Delta {\text{G}}_{\text{com}}-(\Delta {\text{G}}_{\text{ache}}+\Delta {\text{G}}_{\text{inh}})$$ (1)

We utilized data from the prior publication to obtain the computation factors for BFE (Binding Free Energy) [36–38]. These factors were defined as molecular mechanics energy (ΔE_mm_) and solvation free energy (ΔG_sol_), which were calculated using equation (2).


$${\text{E}}_{\text{MM}}={\Delta \text{E}}_{\text{int}}+{\Delta \text{E}}_{vdW}+{\Delta \text{E}}_{\text{ele}}$$ (2)

The investigation focused on breaking down the molecular mechanic’s energy into specific components: van der Waals energies (E*vdW*), non-bonded electrostatic energies (ΔE_ele_), and the solvation free energy (ΔG_sol_). ΔG_sol_, in particular, included both polar and nonpolar solvation energies.


$${\Delta \text{G}}_{\text{sol}}={\Delta \text{G}}_{\text{ele},\text{sol}\;\text{PB}(\text{GB})}+\Delta_{\text{Gnonpol},\text{sol}}$$ (3)

In line with equation (4), the various factors contributing to the breakdown of energies within the inhibitor interaction were analyzed. This breakdown involved determining the decomposition parameters for van der Waals (ΔG*vdW*), electrostatic (ΔG_ele_), polar (ΔG_ele_, _sol_), and nonpolar (ΔG_nonpol_, _sol_) energies. These distinct contributions were investigated by dissecting the Binding Free Energy (BFE) into individual residual components. The analysis of these decomposition factors utilized the same snapshots that were employed for evaluating the Binding Free Energy.


$${\Delta \text{G}}_{\text{inhibitor}-\text{residue}}={\Delta \text{G}}_{vdW}+{\Delta \text{G}}_{\text{ele}}+{\Delta \text{G}}_{\text{ele},\text{sol}}+{\Delta \text{G}}_{\text{nonpol},\text{sol}}$$ (4)

By utilizing MM/GBSA calculations, researchers gain valuable insights into the molecular mechanisms of ligand binding, which can facilitate the development of more effective and specific drugs.

## Results and discussion

### Yield of solvent extractions

In this study, MeOH and DCM extracts were made after collecting and drying the whole plant. Plant powder was extracted thrice in succession by shaking it intermittently in MeOH and DCM for 24, 48, and 72 hours. The extracts were concentrated using a rotary evaporator heated to 37 ℃. Using 500 g of powder, the % yield of *D. inermis* extracts was 2.9% (14.52 g) and 2.72% (13.61 g) for MeOH and DCM, respectively.

### Antibacterial activity

#### Agar well diffusion method (zone of inhibition, ZOI).

**Fig 1:**
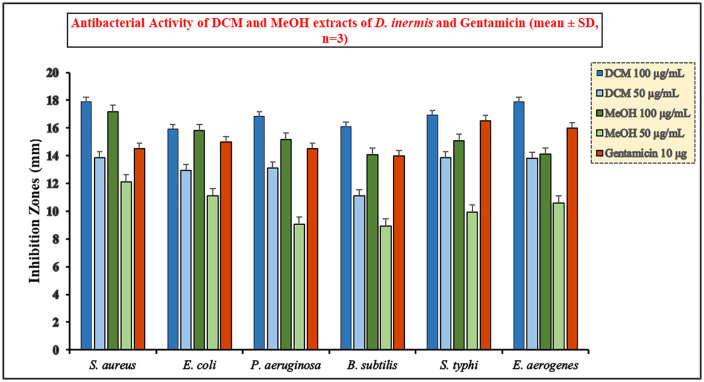
Antibacterial activity of DCM and MeOH extract of *D. inermis* is shown for 100 and 50 µg/mL against *S. aureus*, *B. subtilis*, *E. coli*, *P. aeruginosa*, *S. typhi*, and *E. aerogenes*, compared with gentamicin (10 *µ*g) as a standard antibiotic.

The DCM and MeOH extracts at 100 µg/mL exhibited strong antibacterial activity across all tested strains. Among them, DCM 100 µg/mL showed the highest inhibition zones, with particularly strong effects against *S. aureus* (17.87 ± 0.23 mm) and *E. aerogenes* (17.87 ± 0.23 mm, Susceptible), the latter classified as Susceptible according to CLSI criteria. Other ZOI values for DCM 100 µg/mL included *E. coli* (15.93 ± 0.11 mm, Intermediate), *P. aeruginosa* (16.83 ± 0.29 mm), *B. subtilis* (16.10 ± 0.17 mm), and *S. typhi* (16.93 ± 0.11 mm, Intermediate). The MeOH 100 µg/mL extract also showed substantial antibacterial effects, with highest activity against *S. aureus* (17.17 ± 0.29 mm) and *E. coli* (15.80 ± 0.34 mm, Intermediate), and comparatively lower activity against *B. subtilis* (14.07 ± 0.12 mm), indicating slightly reduced efficacy compared to DCM at the same concentration. At the lower concentration of 50 µg/mL, both extracts retained measurable antibacterial activity. DCM 50 µg/mL showed higher ZOI values than MeOH 50 µg/mL across all strains, ranging from 11.10 ± 0.17 mm (*B. subtilis*) to 13.87 ± 0.23 mm (*S. aureus*), while MeOH 50 µg/mL ranged from 8.93 ± 0.11 mm (*B. subtilis*) to 12.13 ± 0.23 mm (*S. aureus*) [78]. This demonstrates a clear dose-dependent pattern, with antibacterial potency increasing at higher extract concentrations (Fig 1).

Gentamicin 10 µg served as the reference control. ZOI values and SD for each strain are presented in Table 1. Based on CLSI M100 (34th edition) Enterobacterales breakpoints, *E. coli* (15.0 ± 0.12 mm), *E. aerogenes* (16.0 ± 0.12 mm, Intermediate), and *S. typhi* (16.5 ± 0.11 mm) fell into the Intermediate category, while *E. aerogenes* at DCM 100 µg/mL reached the Susceptible category. For *P. aeruginosa* (14.5 ± 0.30 mm), disk-diffusion breakpoints are no longer provided by CLSI, so the value is reported descriptively. *S. aureus* (14.5 ± 0.23 mm) and *B. subtilis* (14.0 ± 0.11 mm) lack CLSI criteria for gentamicin and are presented as raw ZOI values. Overall, the DCM extract demonstrated superior antibacterial activity compared to MeOH at both concentrations, reflecting a higher abundance of active phytoconstituents capable of disrupting bacterial membranes and inhibiting essential enzymes. MeOH extracts, though less potent, still exhibited significant antibacterial effects, indicating bioactive compounds across a broad polarity range. These findings highlight DCM 100 µg/mL as the most promising extract for further phytochemical and molecular docking studies.

**Table 1:** Antibacterial activity of *D. inermis* extracts (mean ZOI mm ± SD) with CLSI categories.

Treatment	*S. aureus*	*E. coli*	*P. aeruginosa*	*B. subtilis*	*S. typhi*	*E. aerogenes*
**DCM 100 µg/mL**	17.87 ± 0.23	15.93 ± 0.11 (I)	16.83 ± 0.29	16.10 ± 0.17	16.93 ± 0.11 (I)	17.87 ± 0.23 (S)
**DCM 50 µg/mL**	13.87 ± 0.23	12.93 ± 0.11 (R)	13.10 ± 0.17	11.10 ± 0.17	13.87 ± 0.23 (R)	13.83 ± 0.29 (R)
**MeOH 100 µg/mL**	17.17 ± 0.29	15.80 ± 0.34 (I)	15.17 ± 0.29	14.07 ± 0.12	15.07 ± 0.10 (I)	14.10 ± 0.18 (R)
**MeOH 50 µg/mL**	12.13 ± 0.23	11.13 ± 0.23 (R)	9.07 ± 0.11	8.93 ± 0.11	9.93 ± 0.11 (R)	10.60 ± 0.18 (R)
**Gentamicin 10 µg**	14.50 ± 0.23	15.00 ± 0.12 (I)	14.50 ± 0.30	14.00 ± 0.11	16.50 ± 0.11 (I)	16.00 ± 0.12 (I)

Note: (S) = Susceptible, (I) = Intermediate, (R) = Resistant according to CLSI M100 (34th ed.) for Enterobacterales only. No CLSI categories exist for *S. aureus*, *B. subtilis*, or *P. aeruginosa* (gentamicin disk breakpoints removed).

### Statistical analysis

**Fig 2:**
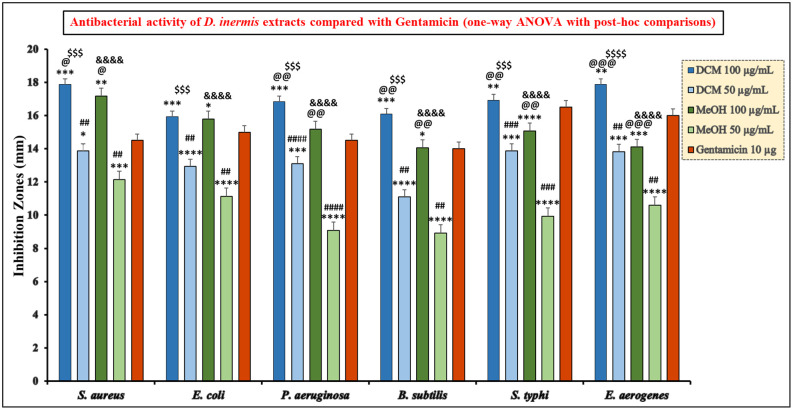
Antibacterial activity of *D. inermis* extracts at 100 and 50 µg/mL compared with Gentamicin (10 µg) against bacterial strains (one-way ANOVA with post-hoc comparisons). Note: One-way ANOVA [* indicates comparison of each extract with Gentamicin (10 µg)]. Pairwise extract comparisons are indicated as: DCM 100 µg/mL vs MeOH 100 µg/mL (@), DCM 50 µg/mL vs MeOH 50 µg/mL (#), DCM 100 µg/mL vs DCM 50 µg/mL ($), and MeOH 100 µg/mL vs MeOH 50 µg/mL (&). The number of symbols denotes the level of significance (*p < 0.05, **p < 0.01, ***p < 0.001).

One-way ANOVA revealed that both DCM and MeOH extracts of *D. inermis* exerted significant antibacterial effects across all tested bacterial strains, as detailed in S1 Table, demonstrating variability in extract potency among different bacteria. Overall, the DCM extract exhibited stronger antibacterial activity, with several strains showing highly significant inhibition (*** to ****). In contrast, the MeOH extract showed moderate activity, with some strains reaching significant inhibition (up to ****), while others, such as *P. aeruginosa* at 100 µg/mL, displayed no significant effect (ns). These findings indicate that the antibacterial efficacy of the MeOH extract is both strain- and concentration-dependent, as illustrated in Fig 2. Pairwise post-hoc analysis revealed distinct differences in antibacterial efficacy between extract types and concentrations. For most bacterial strains, DCM at 100 µg/mL exhibited superior antibacterial activity compared to MeOH at the same concentration, with significant differences observed in *S. aureus* (p = 0.037, *), *P. aeruginosa* (p = 0.002, **), *B. subtilis* (p = 0.0008, **), *S. typhi* (p = 0.0007, **), and *E. aerogenes* (p = 0.00001, ***), as summarized in S2 Table. The strongest inhibition was noted against *E. aerogenes*, while *E. coli* showed no significant difference between the two extracts at this concentration (p = 0.71, ns), suggesting comparable activity at the highest dose. A clear dose-dependent response was observed: raising the concentration from 50 to 100 µg/mL significantly enhanced antibacterial activity for both DCM and MeOH across all tested strains (p < 0.001). This effect was particularly pronounced in *S. aureus* and *E. aerogenes*, where the higher dose produced the most substantial increase in inhibition, confirming that potency is strongly concentration-dependent. At the lower dose, DCM 50 µg/mL consistently outperformed MeOH 50 µg/mL, with highly significant differences in all strains (p < 0.001, #), as illustrated in Fig 2. These results indicate that DCM extracts possess inherently stronger antibacterial activity, even at reduced concentrations, while MeOH also demonstrated notable efficacy, reflecting the presence of bioactive compounds in *D. inermis* with a wide polarity spectrum.

#### Broth microdilution method (minimum inhibitory concentration, MIC).

**Fig 3:**
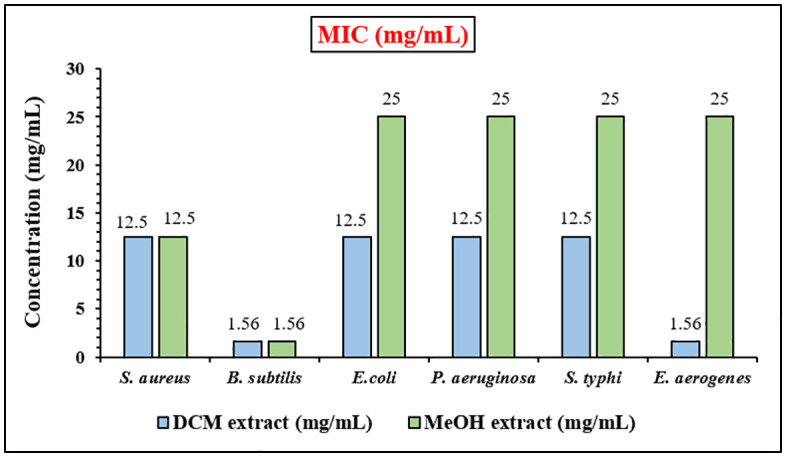
MIC (mg/ml) of DCM and MeOH extract of *D. inermis* against *S. aureus, B. subtilis, E. coli, P. aeruginosa, S. typhi, E. aerogenes.*

The MIC values of the DCM and MeOH extracts of *D. inermis* were evaluated to assess their antibacterial potency against selected bacterial strains. The DCM extract exhibited substantial inhibition across all tested pathogens, with MIC values of *S. aureus* (12.5 mg/mL), *B. subtilis* (1.562 mg/mL), *E. coli* (12.5 mg/mL), *P. aeruginosa* (12.5 mg/mL), *S. typhi* (12.5 mg/mL), and *E. aerogenes* (1.562 mg/mL) (Fig 3). In comparison, the MeOH extract also demonstrated inhibitory activity against all strains; however, the MIC values were generally higher, indicating slightly lower potency. Specifically, inhibition was observed for *S. aureus* (12.5 mg/mL), *B. subtilis* (1.562 mg/mL), while *E. coli*, *P. aeruginosa*, *S. typhi,* and *E. aerogenes* required 25 mg/mL for effective inhibition (Fig 3). These findings, obtained via the microdilution broth method, clearly indicate that the DCM extract possesses superior antibacterial activity compared to the MeOH extract (Table 2). In the broth microdilution assay, vancomycin (20 µL) served as the positive control and exhibited strong antibacterial activity, as indicated by the deep blue–purple coloration across all tested strains, while DMSO, used as the negative control, showed no color change as shown in S1 Fig. This contrast confirms the reliability and validity of the assay and supports the observed antibacterial effects of the DCM and MeOH extracts of *D. inermis*. Both extracts demonstrated significant antibacterial activity, with the DCM extract displaying superior potency; however, the inhibitory effect of the MeOH extract remains notable and cannot be ignored. Consequently, both extracts were selected for further analysis to identify the bioactive compounds responsible for the antibacterial activity of *D. inermis*.

**Table 2:** Growth of *S. aureus, B. subtilis, E. coli, P. aeruginosa, S. typhi, and E. aerogenes* at different concentration of MeOH and DCM extract of *D. inermis* to elucidate MIC.

DCM extract (mg/ml)	*S. aureus* (ATCC 29213)	*B. subtilis* (ATCC 6633)	*E. coli* (ATCC 25922)	*P. aeruginosa* (ATCC 27853)	*S. typhi* (ATCC 14028)	*E. aerogenes* (ATCC 13048)
**50**	No	No	No	No	No	No
**25**	No	No	No	No	No	No
**12.5**	No	No	No	No	No	No
**6.25**	Yes	No	Yes	Yes	Yes	No
**3.125**	Yes	No	Yes	Yes	Yes	No
**1.562**	Yes	No	Yes	Yes	Yes	No
**MeOH extract (mg/ml)**	***S. aureus*** **(ATCC 29213)**	***B. subtilis*** **(ATCC 6633)**	***E. coli*** **(ATCC 25922)**	***P. aeruginosa*** **(ATCC 27853)**	***S. typhi*** **(ATCC 14028)**	***E. aerogenes*** **(ATCC 13048)**
**50**	No	No	No	No	No	No
**25**	No	No	No	No	No	No
**12.5**	No	No	Yes	Yes	Yes	Yes
**6.25**	Yes	No	Yes	Yes	Yes	Yes
**3.125**	Yes	No	Yes	Yes	Yes	Yes
**1.562**	Yes	No	Yes	Yes	Yes	Yes

### Identification of bioactive constituents by GC-MS analysis

**Fig 4:**
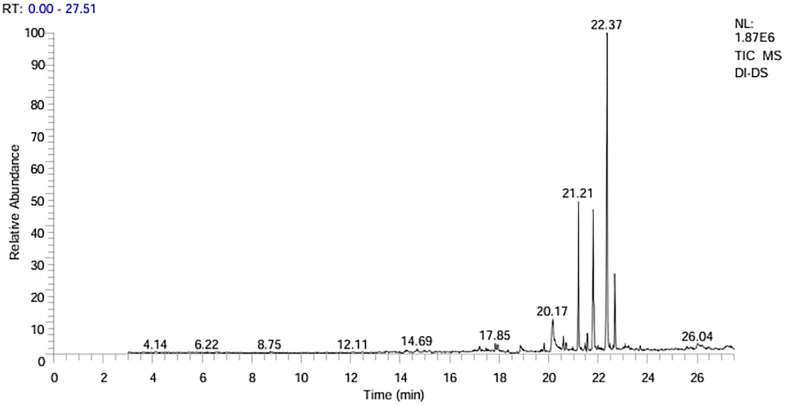
GC-MS chromatogram of DCM extract of *D. inermis.*

GC-MS analysis of the DCM extract from the whole plant of *D. inermis* resulted in the identification of 85 compounds, which are listed in Table 3. Each constituent in the extract was quantified and identified by standards such as replib and MAINLIB library. The chromatogram showed ten peaks with retention time between 4.14 and 26.04 (Fig 6). In the GC-MS analysis, one component representing 99.46% of the total composition was identified, as presented in Fig 4. The major identified compounds were dodecenedioic acid or fatty acids (18.82%), cyclohexanols and alkanes (8.23%), alkaloids (4.70%), terpenoids, monoterpenoids, steroids, sesquiterpenoids, sterols and cannabinoids (32.94%), phorbol ester (9.411%), epoxide (3.529%), isohumolone (3.529%), Phenylhydrazine, Phenylenediamine, Naphthopyran, and brominated farnesyl (4.70%). Other compounds such as ester, piperazine, retinoid, and pregnan glycoside were also identified by GC-MS analysis. Several of the identified compounds have been reported in other *Dipsacus species* [11,79,80], providing supporting evidence for the consistency of our phytochemical profiling. Importantly, some compounds appear to be unique to *D. inermis*, highlighting its distinct chemical profile.

**Table 3:** Total compounds identified by GC-MS analysis.

Sr. no.	Compound Label	RT	PA	PH	Mass	Probability	DB Formula	Chemical class
**1**	3-Ethyl-5-(2’ethylbutyl)octadecane	4.14	12583.25	1608.52	366	12.80	C26H54	Alkane
**2**	9-n-Hexylheptadecane	4.14	12583.25	1608.52	324	11.81	C23H48	Alkane
**3**	n-Tetratetracontane	4.14	12583.25	1608.52	618	9.98	C44H90	Alkane
**4**	(2-Phenyl-1,3-dioxolan-4-yl)methyl-9-octadecenoate	6.63	17185.70	1503.87	444	31.91	C28H44O4	Fatty acid
**5**	Ethyl linoleate	6.63	17185.70	1503.87	308	8.35	C20H36O2	Fatty acid
**6**	Acetic acid, 4-hydroxy-cyclohexyl ester	7.03	13241.01	1223.53	158	4.78	C8H14O3	Cyclohexanols
**7**	(25S)-3Beta-acetoxy-5alpha,22beta-spirost-9(11)-en-12beta-ol	7.03	13241.01	1223.53	472	3.85	C29H44O5	Terpenoids
**8**	2-(1-Methyl-2-nitroethyl) cyclohexanone	7.03	13241.01	1223.53	185	3.70	C9H15NO3	Cylcohexanone
**9**	1, 13-(Acetyloxy) tridecyl acetate	8.81	45462.59	2523.75	300	6.80	C17H32O4	Fatty acid
**10**	8-Acetyl-8-azabicyclo[3.2.1]octane	8.81	45462.59	2523.75	153	5.21	C9H15NO	Alkaloid
**11**	Methyl ester 4-hydroxy octadecenoate	12.11	25595.22	1856.76	314	11.69	C19H38O3	Fatty acid
**12**	3-(Octadecyloxy)propyl (E)-9-octadecenoate	12.11	25595.22	1856.76	592	11.24	C39H76O3	Fatty acid
**13**	2,3-Bis[(E) −9-octadecenoyloxy]propyl (E)-9-octadecenoate	12.45	17969.27	1768.99	884	17.20	C57H104O6	Fatty acid
**14**	10àH-Ambros-11(13)-en-12-oic acid, 6á,15-dihydroxy-4-oxo-, ç-lactone, acetate	12.45	17969.27	1768.99	306	7.83	C17H22O5	Sesquiterpene
**15**	2,7-Diphenyl-1,6-dioxopyridazino[4,5:2’,3’]pyrrolo[4’,5’-d]pyridazine	12.45	17969.27	1768.99	355	5.37	C20H13N5O2	Alkaloid
**16**	2-Nonadecanone, (2,4-dinitrophenyl)hydrazone	13.17	20780.31	1825.07	462	10.03	C25H42N4O4	Phenylhydrazine
**17**	Methyl 3-(acetyloxy)-20-hydroxyurs-12-en-28-oate	13.17	20780.31	1825.07	528	7.28	C33H52O5	Triterpenoid
**18**	2-Cyclopenten-1-one, 3,4-dihydroxy-5-(3-methyl-2-butenyl)-2 -(3-methyl-1-oxobutyl)-4-(4-methyl-1-o xo-3-pentenyl)-	13.17	20780.31	1825.07	362	4.19	C21H30O5	Isohumulone
**19**	Tricyclo-undecan-3-ol, 2-methylene-6,8,8-trimethyl-	13.45	48232.23	3901.15	220	9.11	C15H24O	Sesquiterpenoid
**20**	Caryophyllene oxide	13.45	48232.23	3901.15	220	8.05	C15H24O	Epoxide
**21**	Cedr-8-en-13-ol	13.45	48232.23	3901.15	220	6.00	C15H24O	Isocedrane sesquiterpenoid
**22**	4-[(4-Aminophenyl)sulfonyl]-1,2-benzenediamine	13.76	55993.08	3324.12	263	23.18	C12H13N3O2S	Phenylenediamine
**23**	2,7-Diphenyl-1,6-dioxopyridazino-pyrrolo[4’,5’-d]pyridazine	13.76	55993.08	3324.12	355	12.65	C20H13N5O2	Alkaloid
**24**	Methyl 2-(acetyloxy)-10-(2-hexylcyclopropyl) decanoate	13.76	55993.08	3324.12	368	9.69	C22H40O4	Fatty acid
**25**	Tetracyclo[6.3.2.0(2,5).0(1,8)]tridecan-9-ol, 4,4-dimethy	14.27	86801.78	7458.36	220	11.54	C15H24O	Cyclic alcohol
**26**	Longifolenaldehyde	14.27	86801.78	7458.36	220	5.86	C15H24O	Sesquiterpenoid
**27**	1- Heptatriacontanol	14.69	98204.66	8217.92	536	8.42	C37H76O	Fatty acid
**28**	Epiglobulol	14.69	98204.66	8217.92	222	5.27	C15H26O	Sesquiterpene alcohol
**29**	Isocolumbin	14.69	98204.66	8217.92	358	3.93	C20H22O6	Naphthopyran
**30**	à-Bisabolol	15.16	106113.84	5522.82	222	5.49	C15H26O	sesquiterpenoid
**31**	Farnesyl bromide	15.16	106113.84	5522.82	284	5.49	C15H25Br	brominated farnesyl
**32**	N-(4-Hydroxyphenyl)retinamide	15.16	106113.84	5522.82	391	5.28	C26H33NO2	Retinoid
**33**	5HCyclopropa [3,4]benz[1,2e]azulen-5-one, 3,9,9a-tris(acetyloxy)-3-[(acetyloxy)methyl]-2-chloro-1,1a,1b,2,3,4,4a,7a,7b,8,9,9a-dodecahydro4a,7bdihydroxy1,1,6,8tetramethyl,[1aR(1aà,1bá,2à,3á,4aá,7aà,7bà,8à,9á, 9aà)]	15.47	7259.98	1181.87	584	8.42	C28H37ClO11	phorbol ester
**34**	10àH-Ambros-11(13)-en-12-oic acid, 1,4á,6á,15-tetrahydroxy-, ç-lactone, 15-acetate	15.47	7259.98	1181.87	324	6.45	C17H24O6	Triterpenoid
**35**	5H-Cyclopropa [3,4]benz[1,2-e]azulen-5-one,2,9,9aTris (acetyloxy)-3[(acetyloxy)methyl] 1,1a,1b,2,3,4,4a,7a,7b,8,9, 9a-dodecahydro-3,4a,7b-trihydroxy-1,1,6,8-tetra methyl[1Ar(1aà,1bá,2à,3á,4aá ,7aà,7bà,8à,9á, 9aà)]-	15.47	7259.98	1181.87	584	5.44	C28H37ClO11	phorbol ester
**36**	3-Ethyl-5-(2’-ethylbutyl), octadecane	15.59	13357.59	1630.15	366	8.03	C26H54	Alkane
**37**	1- Oxaspiro[4.4]non-8-ene-4,7-dione	16.22	18256.79	1674.08	362	26.65	C21H30O5	Spiroisohumulone
**38**	2-Cyclopenten-1-one, 3,4-dihydroxy-5-(3-methyl-2-butenyl)-2 -(3-methyl-1-oxobutyl)-4-(4-methyl-1-o xo-3-pentenyl)	16.22	18256.79	1674.08	362	17.21	C21H30O5	Isohumulone
**39**	Olean-12-ene-3,16,21,22,28-pentol, 21-(2-methyl-2-butenoate), [3á,16à,21á(Z),22à]	16.22	18256.79	1674.08	572	5.76	C35H56O6	Triterpenoid
**40**	Digitoxin	16.55	15305.52	1866.81	764	12.93	C41H64O13	Alkaloid
**41**	7,8,12-Tri-O-acetyl ingol	16.55	15305.52	1866.81	492	6.00	C26H36O9	Ingol diterpenoid
**42**	Ethanone, 1-[1-hydroxy-3,3-dimethyl-2-(3-methyl −1,3 butadienyl)cyclopentyl][1à,2à(E)]	17.22	264758.39	14676.95	222	9.64	C14H22O2	Cyclopentanol
**43**	3-Methyl-4-(1,3,3-trimethyl-7-oxa-bicyclo[4.1.0]hept-2-yl)-but-3-en-2-one	17.22	264758.39	14676.95	222	8.89	C14H22O2	Norisoprenoid
**44**	Menthol, 1’-(butyn-3-one-1-yl)-, (1R,2S,5R)	17.22	264758.39	14676.95	222	8.20	C14H22O2	monoterpenoid
**45**	1,2-Epoxyoctadecane	17.52	160711.46	10341.31	268	9.31	C18H36O	Epoxide
**46**	Pyrazole[4,5-b]imidazole 1-formyl-3-ethyl-6-á-d-ribofuranosyl	17.91	505169.96	25177.14	296	30.80	C12H16N4O5	Terpenoid
**47**	4,4,8-Trimethyltricyclo [6.3.1.0(1,5)]dodecane-2,9-diol	17.91	505169.96	25177.14	238	14.03	C15H26O2	sesquiterpenoid
**48**	Picrotoxinine	17.91	505169.96	25177.14	292	13.48	C15H16O6	Alkaloid
**49**	n-Hexadecanoic acid	18.91	326133.78	19833.07	256	45.32	C16H32O2	Fatty acid
**50**	l-(+)-Ascorbic acid 2,6-dihexadecanoate	18.91	326133.78	19833.07	652	17.91	C38H68O8	Fatty acid
**51**	Palmitic anhydride	18.91	326133.78	19833.07	494	13.36	C32H62O3	Fatty acid
**52**	Estra-1,3,5(10)-trien-17á-ol	19.81	112923.94	9591.06	256	13.81	C18H24O	Terpenoid
**53**	Phytol	19.8	112923.94	9591.06	296	10.86	C20H40O	acyclic diterpene alcohol
**54**	4HCyclopropa [5’, 6’]benz [1’, 2’: 7,8]azuleno[5,6-b]oxiren-4-one,8-(acetyloxy)1,1a,1b,1c,2 a,3,3a,6a,6b,7,8,8a-dodecahydro-3a,6b,8a-trihydroxy-2a-(hydroxymethyl)-1,1,5,7-tetramethyl[1ar (1aà,1bá,1cà,2aà,3aá,6aà,6bà,7à,8 á,8aà)]	19.81	112923.94	9591.06	422	6.79	C22H30O8	phorbol ester
**55**	9,12-Octadecadienoic acid (Z,Z)	20.17	1391813.09	105799.99	280	20.66	C18H32O2	Fatty acid
**56**	Ethyl linoleate	20.17	1391813.09	105799.99	308	4.32	C20H36O2	Fatty acid
**57**	4,4,9,9-Tetramethyl-4,9-disilatricyclo[6.2.0.0(3,6)-decane	20.60	305255.85	21554.50	218	24.55	C12H18Si2	Disilatricyclo-decane
**58**	6-Amino-4-(3,4-dichlorophenyl)-3-propyl-1,4-dihydropyrano[2,3-c]pyrazole-5-carbonitrile	20.60	305255.85	21554.50	348	14.13	C16H14Cl2N4O	Ester
**59**	Flunarizine	20.60	305255.85	21554.50	404	11.94	C26H26F2N2	Piperazine
**60**	E,E,Z-1,3,12-Nonadecatriene-5,14-diol	20.97	9877.31	1826.62	294	14.94	C19H34O2	Fatty acid
**61**	ë9-Tetrahydrocannabivarin	21.21	1617897.60	181433.88	286	58.08	C19H26O2	cannabinoid
**62**	Podocarpa-8,11,13-trien-13-ol, 14-isopropyl	21.21	1617897.60	181433.88	286	7.42	C20H30O	Diterpenoid
**63**	Resorcinol, 2-p-mentha-1,8-dien-3-yl-5-pentyl	21.80	2510022.57	223848.45	314	45.43	C21H30O2	benzenediol
**64**	Dronabinol	22.35	6563499.94	585879.30	314	86.94	C21H30O2	cannabinoid
**65**	D-Homo-24-nor-17-oxachola-20,22-dien e-3,16-dione, 7-(acetyloxy)-1,2:14,15:21,23-triepoxy-4,4,8-trimethyl-, (5à,7à,13à,14á,15á,17aà)-	23.15	339854.72	16627.32	498	13.35	C28H34O8	phorbol ester
**66**	Safranin	23.15	339854.72	16627.32	315	9.16	C20H19N4	Quinone e Imine
**67**	Corticosterone	23.15	339854.72	16627.32	346	5.55	C21H30O4	corticosteroid
**68**	Dodecanoic acid, 1a,2,5,5a,6,9 ,10,10a-octahydro-5,5a-di hydroxy-4-(hydroxymethyl)-1,1 ,7,9-tetra methyl-11-oxo-1H-2,8a-methanocyclop enta[a]Cyclo propa[e]cyclodecen-6-yl ester,	23.70	72201.08	5250.29	530	24.89	C32H50O6	Fatty acid
**69**	12-Deoxyphorbol-13-(2-methylpropionate)	23.70	72201.08	5250.29	418	16.58	C24H34O6	phorbol ester
**70**	Pregn-5-ene-3,11,12,14,20 pentol, 11-acetate 12-(3methylbutanoate) (3á,11à,12á,14á)	23.70	72201.08	5250.29	492	6.55	C28H44O7	Pregnane glycoside
**71**	1H-Cyclopropa [3,4]benz[1,2-e]azulene-tetrol-5,7b,9,9a-tetrol, 3-[(acetyloxy)methyl]-1a,1b,4, 4a,5,7a,8,9-octahydro-1,1,6,8-tetr amethyl-,9,9adiacetate,[1aR(1aà ,1bá,4aà,5á,7aà,7bà,8à,9á,9aà)]-	24.02	31599.23	3269.78	476	39.85	C26H36O8	phorbol ester
**72**	3- Hydroxyspirost-8-en-11-one	24.02	31599.23	3269.78	428	5.92	C27H40O4	steroidal sapogenin
**73**	9-Desoxo-9-x-acetoxy-3, 8, 12-tri-O-acetylingol	24.29	13855.02	1549.30	536	11.93	C28H40O10	Ingol diterpenoid
**74**	17-(2-Hydroxy-1,5-dimethyl-hex 4-enyl)-4,4,10,13,14-penta methyl-2,3,4,5,6,7, 10,11,12,13, 14,15,16,17-tetradecahydro-1H-cyclopenta[a]phenanthrene	24.29	13855.02	1549.30	442	7.23	C30H50O2	gorgosterol
**75**	16-Oxapentacyclo [13.2.2.0(1,13) .0(2,1 0).0(5,9)]nonadec-6-ene, 6-acetyloxy-15-ethoxy-5,14,14-trimethyl	24.29	13855.02	1549.30	402	6.66	C25H38O4	steroid
**76**	4H-Cyclopropa [5’, 6’]benz-azuleno[5,6-b]oxiren-4-one	24.57	13774.33	1776.36	422	39.18	C22H30O8	phorbol ester
**77**	4H-Cyclopropa [5’, 6’]benz [1’, 2’: 7,8]azuleno [5,6-b]oxiren −4-one, 8-(acetyloxy)-1,1a,1b, 1c,2a,3,3a,6a,6b,7,8,8a-dode cahydro-3a,6b,8a-trihydroxy-2a-(hydroxymethyl)-1,1,5,7-tetramethyl -, [1ar-(1aà,1bá,1 cà,2aà,3aá,6aà,6bà,7à,8 á,8aà)]	24.57	13774.33	1776.36	420	20.40	C24H36O6	Epoxy androstane
**78**	Vitamin E	25.16	116075.66	3677.70	430	16.81	C29H50O2	tocopherols
**79**	Propanoic acid,2-(3-acetoxy-4, 4,14-trimethylandrost-8-en-17-yl)	25.16	116075.66	3677.70	430	12.20	C27H42O4	gorgosterol
**80**	12-O-Acetylingol 8-tiglate	25.16	116075.66	3677.70	490	11.73	C27H38O8	phorbol ester
**81**	5-Cholestene-3-ol, 24-methyl-	25.63	136512.75	7823.35	400	10.28	C28H48O	phytosterol
**82**	Ethyl iso-allocholate	25.63	136512.75	7823.35	436	6.69	C26H44O5	Steroid
**83**	ç-Sitosterol	26.08	520043.84	23244.73	414	38.34	C29H50O	Steroid
**84**	9,12,15-Octadecatrienoic acid, 2,3-bis[(trimethylsilyl)oxy]propyl ester(Z,Z,Z)	26.77	20885.61	2399.64	496	31.32	C27H52O4Si2	Fatty acid
**85**	D:A-Friedooleanan-3-ol	27.24	228398.53	13739.39	428	13.99	C30H52O	sesquiterpene lactone

**Fig 5:**
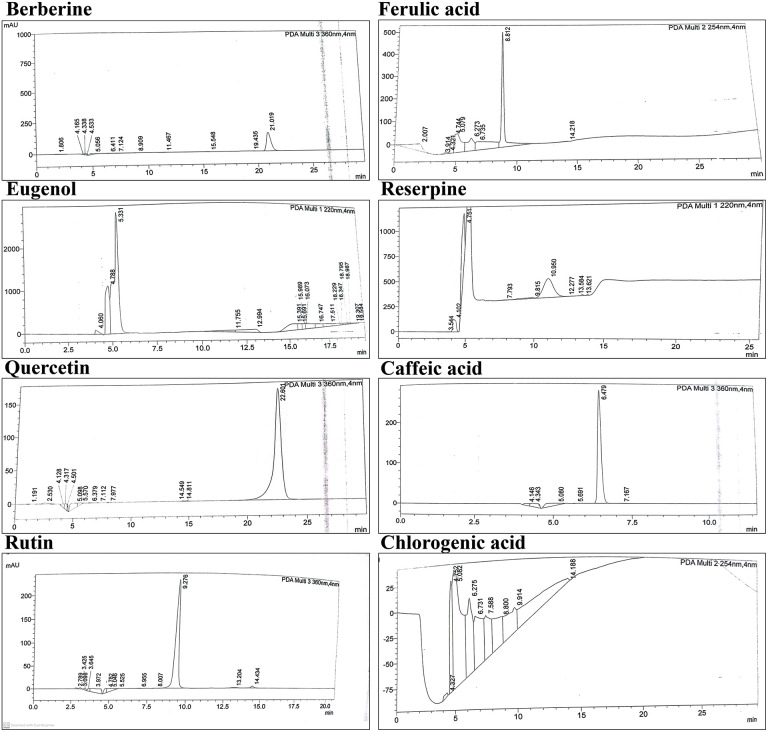
Chromatogram of standards (berberine, ferulic acid, eugenol, reserpine, quercetin, caffeic acid, rutin and chlorogenic acid) obtained by using HPLC-PDA.

### HPLC-UV/Vis analysis

**Fig 6:**
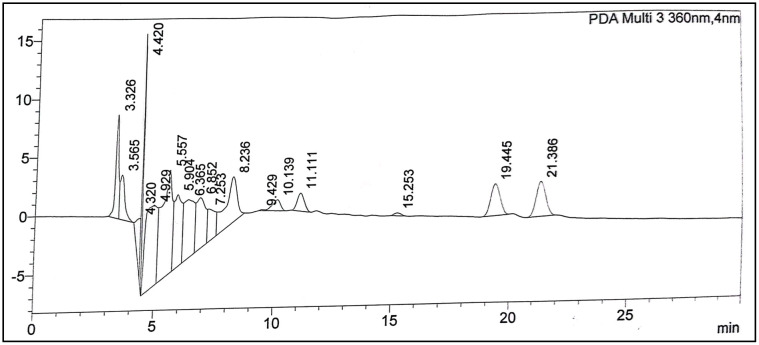
Chromatogram of sample (MeOH extract) obtained by using HPLC-PDA.

To better understand the chemical composition of the fractions, HPLC-UV/Vis analysis was conducted on the methanolic fraction of the fragrant bay tree. In this study, a methanol (MeOH) extract from the whole plant of *D. inermis* was analyzed using HPLC-UV/Vis to quantify the presence of alkaloids, phenolics, and flavonoids, using eight standards as references. Comparative analysis revealed the presence of six compounds (alkaloids, phenolics, and flavonoids) in the tested extracts. The identified and quantified phenolic constituents are presented in Table 4, and the HPLC chromatograms are shown in Figs 5 and 6. The results indicated that D. inermis extracts contained a significant number of phenolic compounds. The MeOH extract showed the highest concentrations of rutin (68.66 *μ*g/mg), reserpine (53.76 *μ*g/mg), and ferulic acid (31.64 *μ*g/mg), while quercetin was below the limit of detection (BLD) (<0.1 *μ*g/mg). These findings align with previous studies on this genus, which reported the presence of compounds such as gallic acid and rutin [81,82]. Overall, the results confirmed the existence of phenolic and flavonoid compounds, suggesting that *D. inermis* can be considered for the isolation of therapeutically important bioactive molecules.

**Table 4:** Retention time (tR), Area under curve (AUC) and concentration of the standards and sample in HPLC- UV/Vis analysis.

Sr. No	St name	AUC of Std	Rt of Std (min)	AUC of sample	Rt of sample (min)	Cal. *µ*g/ml	*µ*g/mg	Conc. In 13.52 g	Conc. In 500 g (*µ*g/500 g)	Conc. (*µ*g/g)
**1**	**Berberine**	10877726	21.01	410017	21.38	7.54	0.29	3790.68	3790.68	7.58
**2**	**Ferulic acid**	27952188	8.812	5643356	8.236	40.38	31.64	419216.33	419216.33	838.43
**3**	**Eugenol**	10708935	14.18	–	–	0.00	0.00	0.00	0.00	0.00
**4**	**Reserpine**	9502252	10.95	2350650	10.139	49.48	53.76	712287.00	712287.00	1424.57
**5**	**Quercetin Dihydrate**	8282220	22.60	37977	–	0.92	0.00	0.00	0.00	0.00
**6**	**Caffeic acid**	21648422	6.479	2339000	6.365	21.61	15.10	200018.46	200018.46	400.04
**7**	**Rutin**	4985386	9.276	2546086	9.429	102.14	68.66	909777.42	909777.42	1819.55
**8**	**Chlorogenic Acid**	53517740	5.331	1786402	5.557	6.68	13.45	178269.99	178269.99	356.54

### *In silico* analysis

#### Docking validation.

**Fig 7:**
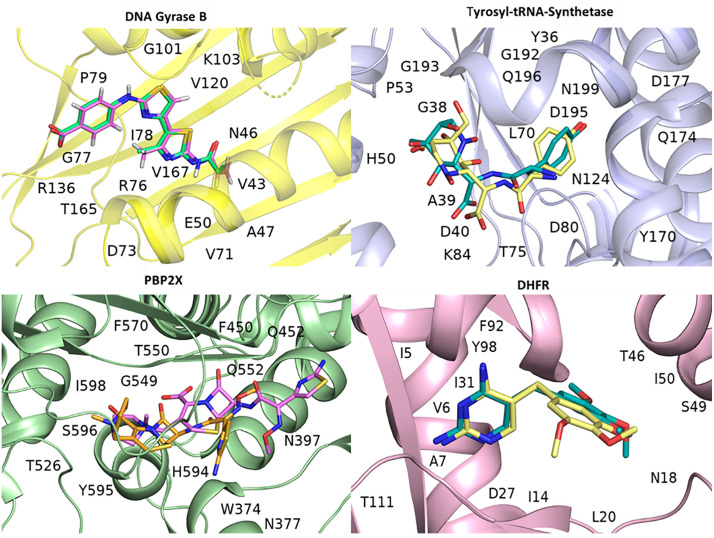
Docking validation of DNA gyrase B, tyrosyl-tRNA-synthetase, PBP2X and DHFR redocked with 4,5’-bithiazole, SB-239629, cefepime and trimethprim, respectively.

To initially validate our docking protocol, 4,5’-bithiazole, SB-239629, cefepime, and trimethoprim were re-docked into the active sites of (DNA gyrase B) 4DUH, (tyrosyl-tRNA synthetase) 1JIJ, (PBP2X) 5OJ0, and (DHFR) 2W9S. The re-docking process was carried out using the Glide XP module of Schrödinger [67], resulting in RMSD values of 1.21 Å. As shown in Fig 9*, docking* almost overlaps the co-crystalized conformation of 4,5’-bithiazole, SB-239629, cefepime, and trimethoprim in 4DUH, 1JIJ, 5OJ0, and 2W9S. These findings affirm the reliability of the protocols in accurately placing back 4,5’-bithiazole, SB-239629, cefepime, and trimethoprim into the ligand-binding pockets of 4DUH, 1JIJ, 5OJ0, and 2W9S, as shown in Fig 7. The docking scores for the 4,5’-bithiazole, SB-239629, cefepime, and trimethoprim confirmation in the Schrödinger protocols were −8.588, −10.913, −6.539, and −9.437 kcal/mol, respectively.

#### Molecular docking.

To assess the binding potential of the selected drug-like compounds identified by GC-MS and HPLC analysis (Codes, Chemical name, and 2D structures given in S3 Table) against DNA gyrase B, tyrosyl-tRNA synthetase, PBP2X, PBP4, and DHFR, all compounds were docked into the active site of proteins. In the case of DNA gyrase B, the binding scores of the compounds ranged from −8.753 to −1.041 kcal/mol, whereas the best compound DI31 scored −8.753 kcal/mol. DI41, DI33, and DI10 had docking scores of-7.562, −6.955, and −6.603 kcal/mol, respectively. The DI31 has highest binding affinity with DNA gyrase B. The tyrosyl-tRNA-synthetase binding scores of the compounds ranged from −7.723 to −1.472 kcal/mol, whereas the best compounds, DI10 and DI31, scored −6.633 and −6.051 kcal/mol, respectively. DI35 demonstrated the highest binding affinity among the analyzed compounds, with a score of −7.723 kcal/mol. DI33, DI22, and DI3 had docking score −6.083, −6.084 and −6.496 kcal/mol respectively, as shown in S4 Table. For PBP2X, the docking scores of the compounds ranged from −8.326 to −2.301 kcal/mol. DI27 demonstrated the highest binding affinity among the analyzed compounds docking score −8.326 kcal/mol. On the other hand, DI10 and DI31 had docking scores of-6.041 and −6.387 kcal/mol, respectively. Apart from other enzymes, DI22 demonstrated the highest binding affinity towards the PBP4 enzyme (−6.625 kcal/mol). DI10 and DI31 had docking scores of-5.556 and −6.631 kcal/mol, respectively, for PBP4, as shown in S4 Table. Finally, for the enzyme DHFR, the docking scores of the compounds ranged from −7.866 to −1.967 kcal/mol. DI10 demonstrated the highest binding affinity among the analyzed compounds docking score −7.866 kcal/mol. On the other hand, DI31 also had the best binding affinity towards the DHFR enzyme (−7.704 kcal/mol). These findings clearly indicate that among the identified compounds, DI10 and DI31 are the main active components responsible for the enzyme inhibitory potential of *D. inermis*. However, compound DI22 is the main compound responsible for the activity against the DHFR enzyme.

**Fig 8:**
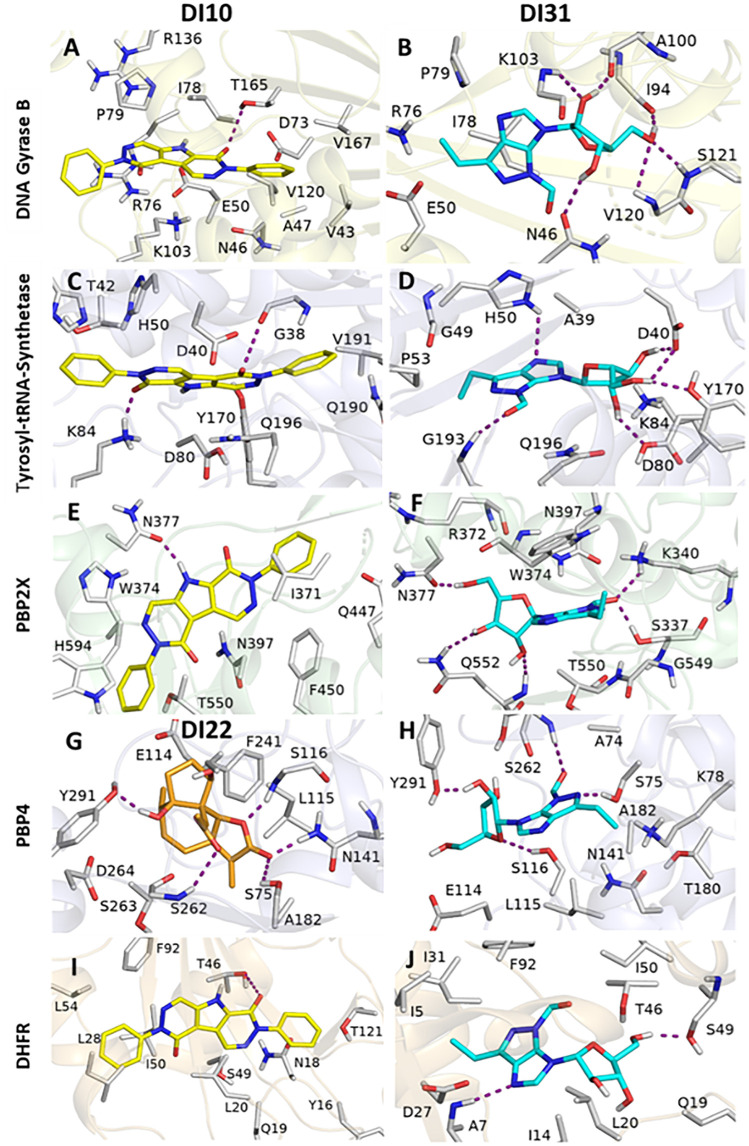
Binding cavity of (A) DNA gyrase B-DI10, (B) DNA gyrase B-DI31, (C) tyrsoyl-tRNA synthetase-DI10, (D) tyrsoyl-tRNA synthetase-DI31, (E) PBP2X-DI10, (F) PBP2X-DI31, (G) PBP4-DI22, (H) PBP4-DI31, (I) DHFR-DI10 and (J) DHFR-DI31.

To elucidate the variances in docking scores resulting from diverse interaction modalities, optimal docking configurations for each molecule were documented and depicted graphically (Fig 8). All ligands, along with benchmark inhibitors, occupy the same binding pocket. In the DNA gyrase B-DI10 complex, the binding involves key residues N46, A47, E50, R76, G77, I78, P79, K103, V120, and T165. DI10 binds tightly through key hydrogen bonds with T165 and enhances hydrophobic interactions via residues R76, P79, I78, K103, and R136, contributing to its specificity and stability (Fig 8 A). DI10 acts by competing with ATP for binding to the structural pocket defined by N46, E50, D73, R76, K103, V120, R136, V167, and T165 in E. coli GyrB [83]. DI10 has an oxygen group at the diazete ring that forms a hydrogen bond with the hydroxyl group extending from T165, as shown in Fig 8 A. Additionally, the diazete ring attaches to the benzene ring, forming a hydrophobic interaction with residues E50 and N46. PLIP analysis showed that DI10 binds tightly to DNA gyrase with key hydrophobic interactions with T165, P79, N46, A47, and V43 with bond lengths range from 3.5 to 3.9, and π-π interactions with K103 aliphatic chains with bond length of 4.3 and 4.4 Å. The hydrogen bonds have bond length range from 3.2 to 4.0 Å as shown in (S2 Fig A). In the DNA gyrase B-DI31 complex, the binding involves key residues N46, E50, R76, I78, P79, I94, A100, K103, V120, S121, and T165. DI31 binds tightly through six hydrogen bonds with N46, I94, A100, K103, S121, and V120, and also enhances hydrophobic interactions via residues R76, P79, I78, and K103, contributing to its binding affinity (Fig 8 B). DI31 penetrates deeply into binding pocket of DNA gyrase subunit B defined by N46, E50, D73, R76, K103, V120 and S121 in *E. coli* Gyrase B. Where DI31 have two hydroxyl group and one methyl-hydroxyl group attach with tetrahydrofuran ring form six hydrogen bonds, the methyl-hydroxyl group of tetrahydrofuran ring makes three hydrogen bonds, each bond with oxygen of I94 and two hydrogen bonds with amino group of V120 and S121. One hydroxyl group extending from the tetrahydrofuran ring forms two hydrogen bonds, each H-bond with the oxygen of A100 and the amine group of K103. Additionally, other hydroxyl groups attach to the tetrahydrofuran ring in the ATP-binding site, forming a hydrogen bond with the oxygen extended from residue N46 [84]. PLIP analysis shows DI31 binds to DNA gyrase with key hydrogen bonds have bond length range from 2.7 to 4.4 Å as shown in (S2 Fig B). Residue K103 have one hydrogen bond with tetrahydrofuran ring with bond length 3.5 Å and residue A100 also have hydrogen bond with tetrahydrofuran ring with bond length 2.7 Å.

In tyrosyl-tRNA synthetase-DI10 binding cavity contains residues Y36, G38, D40, T42, H47, H50, D80, G83, K84, Y170, D177, Q190, G192, and V191. DI10 forms two hydrogen bonds, one hydrogen bond with each residue G38 and K84. The oxygen of both diazete groups of DI10 form one hydrogen bond with the amino group of K84 and the oxygen extended from the main chain residue G38. (Fig 8 C). DI10 binds tightly between the α-helical domain and the long variable arm of tyrosyl-tRNA synthetase, as shown by the residues present in the binding pocket [85]. Using PLIP, Tyrosyl-tRNA synthetase-DI10 hydrophobic interactions with residues Y36 and Q196 in 3.7 Å form a snug pocket around the ligand, facilitating van der Waals interactions as depicted in (S2 Fig C). Binding cavity of tyrosyl-tRNA synthetase-DI10 interactions involve hydrogen bonds by D40 at 4.1 Å, K84 at 2.8 Å, R88 at 3.7 Å, Q174 at 3.8 Å, and H50 at 3.6 Å showing a tight fit and strong interactions in the binding site. H47 forms four salt bridges ranging in 4.4 Å, highlighting precise ionic interactions with DI10. In tyrosyl-tRNA synthetase-DI31 binding cavity have residues A39, D40, G49, H50, P53, D80, K84, Y170, G193 and Q196. DI31 forms six hydrogen bonds, two hydrogen bonds with D40, and one hydrogen bond with residues H50, D80, G193, and Y170. The imidazo [4,5-d] pyrazole group of DI31 forms two hydrogen bonds, one H-bond with the amino group of H50 and one H-bond between the oxygen attached to imidazo [4,5-d] pyrazole and the amino group of G193 (Fig 8 D). DI31 binds tightly between the α-helical domain and long variable arm of tyrosyl-tRNA synthetase, as shown by the residues present in the binding pocket [86]. The hydroxyl groups attached to the tetrahydrofuran ring of DI31 form four hydrogen bonds: one H-bond with the hydroxyl group and oxygen of D40, one H-bond with the hydroxyl group of D80, and two H-bonds with the oxygen and hydroxyl groups of D40 and Y170. PLIP analysis shows DI31 binds to Tyrosyl-tRNA-synthetase with key hydrogen bonds by D40 at 2.8 and 4.0 Å, G193 at 3.8 Å, Y170 at 3.0 Å, Q196 at 3.7 and 3.9 Å and H50 at 3.3 Å showing a tight fit and strong interactions in the binding site as shown in S2 Fig D.

In PBP2X-DI10 have residues W374, N377, N397, F450, T526, Q447, H594 and T550. It has one hydrogen bond with residue N377, as shown in (Fig 8 E). DI10 binds to the active site region (*α*2, *α*6a, *α*8, *β*3, and *β*4 helix) of PBP2X as specified by the residues T550, Q447, and H594 [87]. The pyrrolidine ring between both diazete rings of DI10 forms one hydrogen bond with the oxygen atom of N377, as shown in (Fig 8 E). Using PLIP analysis, we analysed hydrophobic interactions with W374 (3.7 Å and F450 (3.8 Å), as shown in (S1 Fig E). While hydrogen bonds with N397 and N377 measure 3.8 and 2.9 Å respectively. The PBP2X-DI31 binding cavity contained residues S337, K340, R372, W374, N377, N397, G549, T550, Q552, and T550. It has five hydrogen bonds, two H-bonds with Q552, and one H-bond with residues N377, S337, and K340, as shown in (Fig 8 F). The oxygen attached to the imidazo [4,5-d] pyrazole group of DI31 forms two hydrogen bonds: one H-bond with the amino group of K340 and one H-bond with the hydroxyl group of S337. DI31 binds to the active site region (*α*2, *α*6a, *α*8, *β*3, and *β*4 helix) of PBP2X as specified by the residues T550, Q552, and G549 [88]. The hydroxyl group of the tetrahydrofuran ring forms two hydrogen bonds with both the amino groups of Q552, and the methyl-hydroxyl group attaches to the tetrahydrofuran ring with the oxygen of N377, as shown in (Fig 8 F). PLIP analysis shows DI31 binds to PBP2X with key hydrogen bonds by N377 at 2.6 Å, N397 at 3.6 Å, Q552 at 3.1 and 3.2 Å, S337 at 2.8 Å and K340 at 2.9 Å showing a tight fit and strong interactions in the binding site as shown in S2 Fig F.

In PBP4-DI22 bind cavity has residues S75, E114, L115, S116, A182, F241, S262, S263, D264 and Y291. It has five hydrogen bonds, one hydrogen bond with each residue S75, S116, N141, S262, and Y291 (Fig 8 G). DI22 binds tightly to active site residues (mainly S75, F241, Y291, and S262) present between the N-terminus and N-terminal transpeptidase domain [89]. The oxygen attached to the furan ring forms two hydrogen bonds: one hydrogen bond with the hydroxyl group of S75 and the amino group of N141. The hydroxyl group of the cyclopentane ring forms a hydrogen bond with the hydroxyl group extending from Y291. The carboxylic group attached to the furan ring of DI22 forms one hydrogen bond with the amino group of S262. It also has hydrophobic interactions with L115 at 3.7 Å as shown in (S2 Fig G). S75, S116, N141, S262, and Y291 form hydrogen bonds ranging from 2.7 to 3.9 Å that enhance binding specificity. K78 forms four salt bridges ranging in 4.7 Å, highlighting precise ionic interactions with DI22. In PBP4-DI31 bind cavity has residues A74, S75, K78, E114, L115, S116, N141, A182, S262, T180 and Y291. It has four hydrogen bonds, one hydrogen bond with each residue S75, S116, S262, and Y291 (Fig 8 H). DI31 binds tightly to active site residues (mainly S75, S116, F241, Y291, and S262) present between the N-terminus and N-terminal transpeptidase domain [90]. The oxygen attached to the imidazo [4,5-d] pyrazole group of DI31 forms a hydrogen bond with the amino group of S262. The nitrogen in the pyrazole ring formed a hydrogen bond with the hydroxyl group of S75. It also has a hydrophobic interaction with A182 at 3.5 Å as shown in (S2 Fig H). While S75, S116, N141, S262 and Y291 form hydrogen bonds ranging from 2.7 at 4.0 Å that enhance binding specificity of DI31 to PBP4.

In the DHFR-DI10 complex, the binding involves key residues Y16, N18, Q19, L20, L28, T46, S49, I50, L54, F92, and T121. DI10 binds tightly through key hydrogen bonds with T46 and enhances hydrophobic interactions via residues N18, F92, I50, and L28, contributing to its specificity and stability (Fig 8 I). DI10 penetrates deeply into the M20 loop (12–24 residues) and substrate binding pocket (25–55) of DHFR and inhibits the conversion of dihydrofolic acid into tetrahydrofolic acid by binding to the active site defined by Y16, N18, L20, L28, L54, S49, and I50 in *S. aureus* DHFR [91]. DI10 has an oxygen group at the diazete ring that forms a hydrogen bond with the hydroxyl group extending from T46. PLIP analysis showed that DI10 binds tightly to DHFR with key hydrophobic interactions with L28, L54, F92, L20, and T121 with bond lengths of 3.5, 3.5, 3.8, 3.6 and 3.5 Å, and hydrogen bonds with bond lengths range from 3.3 and 3.7 Å with T46 and F92, respectively, as shown in (S2 Fig I). In the DHFR-DI31 complex, the binding involves key residues I5, A7, I14, Y16, N18, Q19, L20, D27, L28, I31, T46, L54, S49, I50, and F92. DI31 forms two hydrogen bonds with S49 and A7 and also enhances hydrophobic interactions *via* residues T42, F92, L20, and I14, contributing to its binding affinity (Fig 8 J). DI31 binds to the M20 loop (12–24 residues) and substrate binding pocket (25–55) of DHFR [92]. In DI31, one methyl-hydroxyl group attached to the tetrahydrofuran ring formed one hydrogen bond with the hydroxyl group of S49. The nitrogen within the pyrazole ring forms a hydrogen bond with the amino group of the residue A7. PLIP analysis showed that DI31 binds tightly to DHFR with key hydrophobic interactions with A7, I5, I31, F92, L54, F92, L20, and T121 with bond lengths range from 3.3 to 5.4 Å, and hydrogen bonds with bond lengths ranging from 3.3, 3.7, 2.9 and 4.0 Å with A7, I14, T46, and S49, respectively (S2 Fig J). Overall, the GCMS-identified compounds from the DCM extract (DI1–DI50) exhibit higher docking scores across all target proteins compared to the HPLC-identified compounds (DIH1–DIH6), indicating stronger predicted interactions and supporting the selection of lead compounds from the DCM extract.

### Structural interaction fingerprint (SIFt) analysis

**Fig 9:**
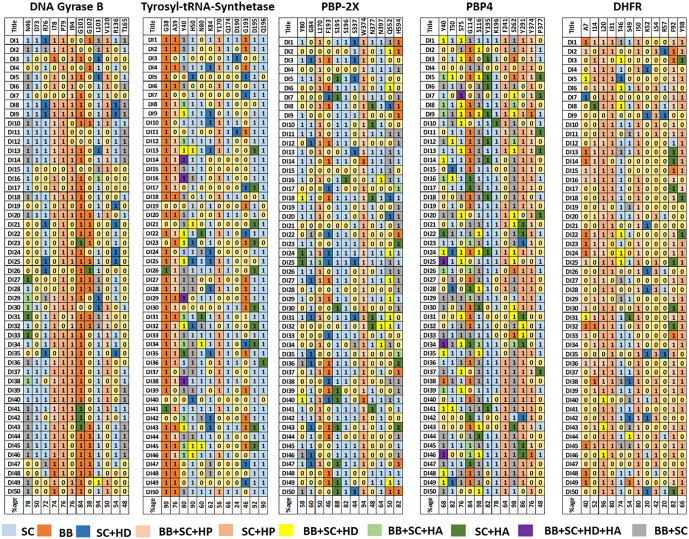
Structural fingerprinting analysis of fifty docked compounds with DNA gyrase B (A), tyrosyl-tRNA synthetase (B), PBP2X (C), PBP4 (D) and DHFR (E).

The structural interaction fingerprint is one of the first and most well-known fingerprints. It is a binary fingerprint, the interaction type is any contact, hydrophobic, aromatic and a hydrogen bond donor or acceptors shown in Fig 9. Contact, Backbone, Side chain, and hydrophobic interactions in each protein were displayed in the light π-acceptor, as shown in Anycast plus backbone and a, such intact plus side chain interaction are shown in orange (common in DNA gyrase B FP analysis) and sky-blue (common in tyrosyl-tRNA synthetase and PBP-2X FP analysis), respectively. Any contact plus backbone plus side chain interaction displayed in grey color. Most importantly, interactions between any contact plus backbone plus side chain plus hydrogen bond donor and any contact plus backbone plus side chain plus hydrogen bond acceptor are displayed in mustard yellow and light green, respectively. Navy blue represents any contact plus side chain plus hydrogen bond donor interactions, as shown in Fig 9. Dark green was also shown important interactions like any contact plus side chain and hydrogen bond acceptor. Finally, any contact plus sidechain plus hydrophobic interaction is shown in a slightly dark pink colour, common in DNA gyrase B and DHFR finger printing analysis, as shown in (Fig 9A and 9E). Finally, the absence of every interaction of compounds with any residue is shown in light yellow colour and mentioned as 0.

Residues present in the binding cavity of DNA gyrase B were N46, D73, R76, I78, P79, I94, G101, G102, K103, V120, and T165 (Fig 9 A). These are the same residues that are present in the binding cavity of DNA gyrase B-DI10 and DNA gyrase B-DI31 because they are essential for residual interaction. Residues G101 and K103 interact with 84 and 94% of compounds docked with DNA gyrase B. DI-27, DI31, and DI-33 have side chain and hydrogen bond acceptor interactions with N46, as shown in dark green. Residues D73 and V120 interacted with 50% of the compounds. Residue G102 interact with least number of compounds (38%) as compare to other residues. Residues N46, R76, I78, P79 and I94 have interaction range within 72–78% of compounds. Dark green indicates any contact plus side chain and hydrogen bond acceptor between residue G101 and compounds DI23 and DI41 to DI43. Navy blue colour represents any contact plus side chain plus hydrogen bond donor interactions present most commonly between residue R76, K103, and R136 and compounds DI3, DI5, DI8, DI9, DI13, DI19, DI21, DI25, and DI35. Most importantly, interactions between any contact plus backbone plus side chain plus hydrogen bond donor, shown in yellow, are common between residue K103 and compound DI49, as shown in Fig 9 A. Mainly orange colour shows backbone interaction with all compounds and residues G101 and G102.

Residues present in tyrosyl-tRNA synthetase-DI10 and DI31 binding cavity are Y36, A39, G38, D40, G49, H50, P53, F54, D80, K84, Y170, Q190, G192, G193, and Q196. These residues G38, A39, D40, T42, H50, T75, D80, K84, Y170, Q174, D195, G193, Q190, and Q196 are also present in fingerprinting analysis, as shown in (Fig 9 B). In the tyrosyl-tRNA-synthetase binding cavity, residues D195, Q196, G38, and H50 interacted with 92, 90, 90%, and 90% of docked compounds, respectively. Residue G190 and Y173 interacted with almost 46 and 56% of docked compounds, respectively. Residues A39 and D40 interacted with 76 and 80%, respectively, of compounds docked with tyrosyl-tRNA synthetase. DI14, DI16, DI29, and DI38 had hydrogen bond acceptor and donor interactions with residue D40, as shown in purple. In tyrosyl-tRNA synthetase FP analysis, backbone interaction in orange colour was most common between G38 and all compounds, as shown in (Fig 9 B). Residue A39 also have backbone interaction shown in orange color and backbone, side chain, hydrophobic interaction with all compounds shown in light pink color. Side chain interaction displayed in sky blue colour most common in residues H50, K84, Q174, D195, and Q196 with approximately all compounds. Residue Y170 mostly has contact plus sidechain plus hydrophobic interaction shown in a slightly dark pink colour with approximately 56% of docked compounds. D80 and D195 also have any contact plus side chain and hydrogen bond acceptor shown in dark green with compounds DI13, DI17, DI21, DI23, DI26, DI30, DI32, DI34, DI43, and DI45.

In the binding cavity of PBP2X, the most interacting residues are Y80, G84, L170, F193, S195, S96, S337, W374, N377, N397, Q552, and H594. These residues are also present in the binding cavities of PBP2X-DI10 and PBP2X-DI31 (S337, R372, W374, N377, N397, F450, Q552, and H594). In the PBP2X binding cavity, residues S195 and W374 interacted with 88 and 94% of compounds, respectively. Moreover, residues S337, G84, and N397 interacted with approximately 44, 60%, and 64% of docked compounds, respectively. Residue F193 and N377 interact with a smaller number of compounds 46 and 48%, respectively as compare to other residues. Residue L170 and Q552 interact with 50% of docked compounds as shown in Fig 9 C. Residue Y80, G84, and N397 interact with 58, 60 and 64% of docked compounds with PBP2X. Residue G84, F193, S337 and Q552 have any contact plus side chain plus hydrogen bond donor interactions with compounds DI1, DI3. DI5, DI7, DI8, DI12, DI13, DI17, DI18, DI20, DI22, DI24, DI25, DI35, DI36, DI38, DI40, DI43, DI46, DI47 and DI50 as shown in navy blue color. F193 mostly has backbone interactions with all docked compounds, shown in orange. W374 has both side chains plus hydrophobic and Backbone, Side chain and hydrophobic interactions shown in slightly dark pink and light pink colour, respectively, with most compounds shown in (Fig 9 C). Side chain interaction is displayed in sky blue colour, most common in residues S195 and S196 with approximately all compounds. D80, S195, S196 and N377 also have any contact plus side chain and hydrogen bond acceptor shown in dark green with compounds DI5, DI7, DI8, DI10, DI16, DI18, DI23, DI24, DI25, DI30, DI31, DI39, DI40, DI41, DI43, DI47, DI49 and DI50.

The PBP4 binding cavity has the most interacting residues Y40, T50, S75, E114, S116, Y195, K196, F241, S262, Y291, Y374, and P377. These residues were also present in the PBP4-DI22 and PBP4-DI31 bind cavity (residues A74, S75, K78, E114, L115, S116, N141, T180, A182, F241, S262, S263, D264, and Y291). Residue T50, E114, Y291, S116 and S262 interact with 82, 84, 86, 98 and 98% of docked compounds with PBP4. Residue P377 interacts with a smaller number of compounds (48%) than other residues in the binding cavity of PBP4. Residue Y40 and F241 interact with 68 and 64% of docked compounds. Residue S75, K196 and Y374 interact with approximately 76–78% of compounds docked in PBP4. In PBP4 FP analysis, residues Y291 and Y374 interacted with approximately all compounds by Backbone, Side chain, and hydrophobic interactions, as shown in light pink, and side chain plus hydrophobic interactions, as shown in slightly dark pink (Fig 9 D). Y40, T50, S75, S116, S262, and Y291 interacted with different compounds with backbones plus side chains plus hydrogen bond donors, as shown in yellow, and approximately 50% of the compounds interacted with backbones plus side chains, as shown in grey. Residue E114 and S262 have backbone interaction with 60% of compounds as shown in orange color. DI25, DI34, and DI46 had both hydrogen bond donor and hydrogen bond acceptor interactions with residue Y40, and DI7 also had both hydrogen bond donor and hydrogen bond acceptor interactions with residue S75, as shown in purple. E114, Y195, Y291 and P377 also have any contact plus side chain and hydrogen bond acceptor shown in dark green with compounds DI3, DI5, DI6, DI7, DI9-DI14, DI16-DI18, DI20-DI22, DI24, DI28, DI30, DI31, DI34, DI37 and DI41 to DI47. Fingerprinting analysis concluded that all the ligands were not only similar in shape, but also had similar electrostatic patterns or interactions with each protein.

Most interacting residues present in the fingerprinting analysis of DHFR were A7, I14, L20, D27, L28, I31, T46, S49, I50, K52, I54, R57, F92, and Y98 (Fig 9 E). These are the same residues that are present in the binding cavity of DHFR-DI10 and DHFR-DI31 (I5, A7, I14, Y16, N18, Q19, L20, D27, L28, I31, T46, I50, L54, S49, I50, F92, and T121) because they are essential for residual interaction. Residues L20, I31, I50 and F92 interact with 96, 80, 80 and 82% respectively of compounds docked with DHFR. Residues I14, S49, F92 and Y98 have side chain and hydrogen bond acceptor interactions with compounds DI3, DI8, DI13, DI14, DI17, DI18, DI22, DI25, DI30 to DI32 and DI50 as shown in dark green color. Residues I14 and S49 interact with 52 and 54% of docked compounds, respectively. Residue R57 and K52 interact with the least number of compounds (20%) as compared to other residues in the binding cavity of DHFR. Residues T46 and Y98 have interaction range within 74 and 66%, respectively docked compounds. Dark green indicates any contact plus side chain and hydrogen bond acceptor between residue F92 and compounds DI13, DI14, DI17, DI25, DI30, DI32, and DI50. Navy blue color represents any contact plus side chain plus hydrogen bond donor interactions present most commonly between residue (T46, K52, and R57) and compounds DI2, DI4, DI5, DI9, DI21, DI26, DI28, DI35, DI38, DI42, and DI50. Most importantly, any contact plus backbone plus side chain plus hydrogen bond donor shown in yellow common between residue T46 and compounds DI3, DI6, DI8, DI22, DI24, and DI25 as shown in Fig 9 E. On the other hand, residue L20 interacts with compounds DI40, DI43, and DI46 by any contact plus backbone plus side chain plus hydrogen bond donor shown in yellow. Mainly orange color shown backbone interaction between compounds (DI1, DI13, DI14, DI22, DI23, DI24, DI32, DI45, DI48 and DI49) and residue A7. Residue L20, I31, I50 and Y98 interact with approximately all compounds by Backbone, Side chain and hydrophobic interactions shown in light pink color and Side chain plus hydrophobic interactions in slightly dark pink.

#### ADMET.

Among the identified compounds, the compound with the highest synthetic accessibility had a value of 7.46, which corresponded to DI-28. Conversely, the compound with the lowest synthetic accessibility had a value of 2.32, attributed to DI-41. To evaluate the *in silico* ADMET properties of these molecules, the pkCSM online tool (https://biosig.lab.uq.edu.au/pkcsm/prediction) was used. When a drug is administered orally, its primary site of absorption is typically the digestive tract. The extent to which a substance is taken up by the human gastrointestinal tract is influenced by various parameters related to intestinal absorption. When the absorption rate of a molecule falls below 30%, it is considered to exhibit inadequate absorption efficacy (https://biosig.lab.uq.edu.au/pkcsm/theory). Compounds DI1 to DI11, DI14, DI15, DI18 to DI20, DI24, DI28 to DI30, DI33, DI35 to DI42 and DI46 to DI50 exhibit high levels of intestinal absorption, indicating they might effectively pass through the intestines into the bloodstream. On the other hand, several compounds such as DI25, DI27, DI31, and DI34 show relatively lower levels of intestinal absorption, suggesting they might have limitations in their ability to pass through the intestinal barrier and get absorbed into the body’s circulation. Table 4 presents the predicted outcomes for these compounds in terms of their ability to inhibit a specific isoform of cytochrome P450. Cytochrome P450 enzymes play crucial roles in the processing of several drugs. However, inhibitors of P450 enzymes can significantly alter the metabolism of these compounds, thereby affecting their pharmacokinetics. Therefore, it is important to consider that the chemicals provided are likely to be cytochrome P450 substrates. Among the various isoforms, cytochrome P450 3A4 is primarily responsible for drug metabolism. The results in the table indicate whether the substances listed will undergo metabolism by cytochrome P450. The clearance of a drug, which involves both hepatic clearance (metabolism in the liver and excretion via bile) and renal clearance (excretion via the kidneys), was evaluated using the proportionality constant CLtot. Bioavailability is a crucial factor in determining the appropriate dosage rates to achieve steady-state concentration. Table 5 also presents the logarithmic values of the expected total clearance (CLtot) of a drug measured in (mL/min/kg). The AMES toxicity test is commonly employed to assess the potential of a compound to induce bacterial mutations.

**Table 5:** ADMET prediction of fifty docked compounds.

Compounds	Pharmacokinetics											Synthetic accessibility
	Absorption	Distribution	Metabolism							Excretion	Toxicity	
	Intestinal absorption	VDss	CYP							Total Clearance	AMES	
	(Human)	(Human)									Toxicity	
	% absorbed	Log L/kg	2D6 Sub	3A4 Sub	1A2 Inh	2C19 Inh	2C9 Inh	2D6 Inh	3A4 Inh	log ml/min/kg	Y/N	Numeric
DI1	90.063	0.153	N	Y	N	N	N	N	N	1.517	N	5.03
DI2	92.782	0.12	N	Y	Y	N	N	N	Y	2.08	Y	5.03
DI3	96.79	-0.117	N	N	N	N	N	N	N	1.313	N	2.57
DI4	97.407	0.197	N	Y	N	N	N	N	N	0.281	N	6.95
DI5	96.346	-0.052	N	N	Y	N	N	N	N	0.488	N	2.9
DI6	94.062	-0.006	N	Y	N	N	Y	N	N	1.838	N	2.57
DI7	95.192	0.256	N	N	N	N	N	N	N	1.275	N	2.63
DI8	91.548	-0.16	N	N	N	N	N	N	N	1.953	N	3.65
DI9	100	0.102	N	Y	N	N	N	N	N	1.172	N	4.3
DI10	100	0.12	N	Y	Y	N	N	N	N	0.207	N	2.66
DI11	97.029	0.032	N	Y	N	Y	N	N	N	1.37	N	4.99
DI12	81.025	-1.5	N	N	Y	N	N	N	N	-0.447	N	6.52
DI13	66.176	-0.345	N	Y	N	N	N	N	N	1.532	N	4.95
DI14	95.338	0.406	N	Y	N	N	N	N	N	0.541	N	3.88
DI15	95.999	0.578	N	N	Y	Y	Y	N	N	0.905	N	4.35
DI16	75.211	-0.025	N	N	N	N	N	N	N	0.371	N	2.36
DI17	93.7	0.578	N	Y	N	N	N	N	N	0.96	N	4.24
DI18	94.952	0.389	N	N	N	N	N	N	N	0.817	N	3.58
DI19	98.23	0.047	N	Y	N	N	N	N	N	0.782	N	5.37
DI20	90.962	0.558	N	Y	Y	N	N	N	N	1.091	N	5.37
DI21	86.972	0.869	N	Y	N	N	N	N	N	0.306	N	6.51
DI22	77.061	0.03	N	N	N	N	N	N	N	1.185	N	4.43
DI23	70.962	0.959	N	N	N	N	N	N	N	0.383	N	6.43
DI24	100	-0.29	N	Y	N	N	N	N	N	1.287	N	5.54
DI25	66.802	-0.214	N	N	N	N	N	N	N	1.552	N	4.87
DI26	77.313	-0.179	N	Y	N	N	N	N	N	0.154	N	7.16
DI27	68.507	0.043	N	Y	N	N	N	N	N	0.429	N	8.74
DI28	92.341	0.235	N	N	N	N	N	N	N	0.747	N	7.46
DI29	94.697	0.066	N	N	N	N	N	N	N	1.389	N	3.93
DI30	97.395	0.034	N	N	N	N	N	N	N	1.23	N	4.89
DI31	68.156	-0.67	N	N	N	N	N	N	N	0.889	N	4.14
DI32	88.171	0.446	N	Y	N	N	N	N	N	0.892	N	5.05
DI33	93.375	1.259	N	Y	Y	Y	N	N	N	0.946	N	3.37
DI34	52.097	0.439	N	N	N	N	N	N	Y	0.576	N	5.83
DI35	92.296	0.247	N	Y	Y	Y	N	N	N	0.71	N	4.08
DI36	91.412	2.124	Y	Y	Y	N	N	Y	N	0.838	N	3.15
DI37	92.018	0.752	N	Y	Y	Y	N	N	N	0.912	N	4.05
DI38	92.137	0.554	N	N	Y	N	N	N	N	1.197	N	4.05
DI39	93.11	0.811	N	Y	Y	Y	N	N	N	0.981	N	4.27
DI40	100	0.044	N	Y	N	N	N	N	Y	0.094	N	6.43
DI41	94.37	-0.28	Y	Y	Y	Y	Y	Y	Y	-0.233	N	2.32
DI42	98.576	-0.098	N	Y	N	N	N	N	N	0.68	N	5
DI43	84.189	-0.429	N	Y	N	N	N	N	N	0.704	N	7.41
DI44	73.156	-0.145	N	N	N	N	N	N	N	0.506	N	5.77
DI45	80.521	0.323	N	N	N	N	N	N	N	0.954	N	6.24
DI46	94.497	0.274	N	Y	N	N	N	N	N	0.501	N	6.21
DI47	97.885	0.375	N	Y	N	N	N	N	N	0.252	N	6.7
DI48	91.179	0.261	N	Y	N	N	N	N	N	0.637	N	4.74
DI49	99.492	0.115	N	Y	N	N	N	N	N	0.621	N	6.25
DI50	98.828	0.162	N	Y	N	N	N	N	N	0.744	N	5.39

#### DFT and MESP studies.

**Fig 10:**
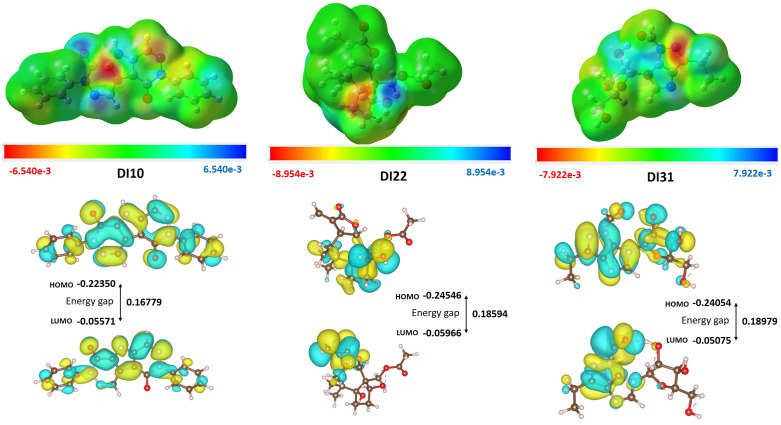
ESP structures (in solvent phases) formed by mapping of total density over electrostatic potential, and optimized structures of DI10, DI22 and DI31. HOMO and LUMO orbitals of potent derivatives of DFT calculations for all selected ligands.

The Molecular Electrostatic Potential (MESP) mappings have conducted a comparative analysis of the electronic properties of inhibitors of DNA gyrase B, tyrosyl-tRNA synthetase, PBP2X, PBP4, and DHFR, specifically targeting compounds DI10, DI22, and DI31. As illustrated in Fig 10, these mappings delineate the electronic characteristics that facilitate biochemical interactions with catalytic residues. In this context, the HOMO-LUMO analysis provides insight into the chemical reactivity and stability of these compounds. Higher HOMO energies reflect electron-donating regions that can form key interactions within enzyme active sites, while lower LUMO energies indicate electron-accepting ability important for interacting with electrophilic residues. The combined MESP and HOMO-LUMO profiles indicate that these compounds possess favorable charge distribution and reactivity to effectively disrupt essential enzymatic functions, thereby supporting their potential antibacterial activity. This crucial analysis is key to assessing the reactivity of compounds. The HOMO-LUMO energy gap, which reflects a molecule’s kinetic stability, facilitates enhanced energy transfer within the molecule. The molecular surface plots of the HOMO and LUMO for DI10, DI22, and DI31 are presented in Fig 12. The LUMO value represents the electron-acceptor potential of an inhibitor, whereas the HOMO value determines its electron-donating ability. S5 Table provides a concise summary of the quantum chemical descriptors calculated for these compounds under aqueous conditions. DFT calculations have clarified crucial molecular characteristics of these DNA gyrase, tyrosyl-tRNA synthetase, PBP2X, PBP4, and DHFR inhibitors, unveiling their distinct electronic structures and reactivity. Using Koopman’s theorem, the global reactivity parameters for ligands DI10, DI22, and DI31 were computed, demonstrating notable differences in their electronic properties, as depicted in S5 Table.

**Fig 11:**
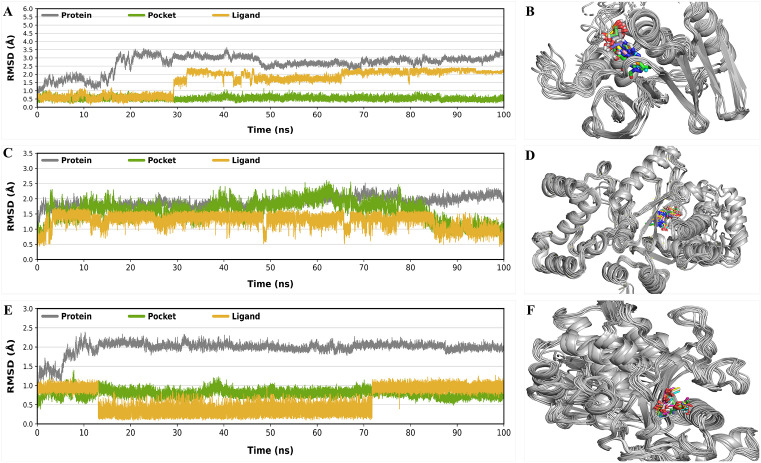
Root-mean-square deviation (RMSD) curve for complex: (A) DNA gyrase B-DI31, (C) Tyrsoyl-tRNA synthetase-DI31 (E) PBP4-DI22 (B) Selected complexes occupying binding cavity in DNA gyrase B with DI31, (D) Selected complexes occupying the same binding site of tyrsoyl-tRNA synthetase with DI10 (F) Selected complexes occupying the same binding site of PBP4 with DI22.

The MESP mappings underscore the areas of pronounced electronegative potential, identified by a deep red hue across all four compounds. These crucial zones indicate preferred sites for electrophilic attack, essential for robust molecular binding and protein inhibition. Detailed Mullikan population analysis revealed that the nitrogen atom at position 11 in DI10 possesses an average Mullikan charge of −0.759904, marked as the most intense red region on the MESP mapping. The rest of the area marked in green can be proven as a crucial area suitable for hydrophobic interaction formation. A similar MESP pattern is observed in rest of the three compounds DI10, DI22 and DI31. This pivotal analysis is essential for determining the reactivity of compounds. The HOMO-LUMO energy gap, which reflects a molecule’s kinetic stability, highlights the energy difference between the highest occupied and lowest unoccupied molecular orbitals, facilitating enhanced energy transfer within the molecule. Molecular surface plots of the HOMO and LUMO orbitals for DI10, DI22, and DI31 are presented in Fig 10. The LUMO value indicates the electron-acceptor potential of an inhibitor, whereas the HOMO value determines its electron-donating capacity. DFT calculations have provided key insights into the molecular characteristics of these DNA gyrase, tyrosyl-tRNA synthetase, PBP2, PBP4, and DHFR inhibitors, revealing their unique electronic structures and reactivity.

DI10, in particular, displayed an exceptionally high dipole moment of 4.284 Debye, presenting a unique electronic profile. DI22 and DI31 also exhibited comparable dipole moments of 10.432 and 4.21 Debye, respectively. In contrast, DI31 and DI10 exhibited exceptionally lower dipole moment (4.21 and 4.284 D) than DI22. This differentiation suggests varied electron-donating capacities and interaction potentials with DNA gyrase, tyrosyl-tRNA synthetase, PBP2X, PBP4, and DHFR. The HOMO-LUMO energy gap analysis further showed that all structures, such as DI10, DI22, and DI31, showed low structural similarity and considerable differences in the HOMO-LUMO gaps, dipole moments, and other electrochemical parameters shown in the table which could be responsible for the different binding patterns of these compounds to the respective proteins. These insights not only enhance our comprehension of the molecular basis for inhibitory activity against microbial proteins but also underscore the imperative to fine-tune electronic and structural attributes for developing potent DNA gyrase, tyrosyl-tRNA synthetase, PBP-2X, PBP4, and DHFR inhibitors. This comprehensive analysis lays the groundwork for future structure-activity relationship studies, which will inform the development of novel inhibitors with enhanced therapeutic profiles.

Using Koopman’s theorem, the global reactivity parameters for ligands DI10, DI22, and DI31 were computed, demonstrating notable differences in their electronic properties, as depicted in the data. DI10 is characterized by a moderate level of electronegativity (χ = 0.13961 eV) and chemical potential (μ = 0.13961 eV), paired with a relatively lower hardness (η = 0.08389 eV). This level of hardness suggests a softer resistance to the deformation of its electron cloud, which is also indicated by its higher global softness (S = 11.91997 eV) and moderate electrophilicity index (ω = 0.11602 eV). In contrast, DI22 exhibited higher electronegativity (χ = 0.15256 eV) and chemical potential (μ = −0.15256 eV), coupled with the highest hardness (η = 0.0929 eV) observed among the studied ligands. This pronounced hardness underscores a more robust resistance to electronic reconfiguration, paired with a lower global softness (S = 10.76482 eV), which correlates with a lower capacity to attract additional electrons, as reflected by its electrophilicity index (ω = 0.12536 eV). DI31 shows a slightly higher electronegativity (χ = 0.14565 eV) and chemical potential (μ = −0.14565 eV) than DI10, with a hardness (η = 0.0949 eV) that suggests a lower resistance to electronic deformation. Its global softness (S = 10.53678 eV) indicates a higher capacity for electron mobility, which is accompanied by a significant electrophilicity index (ω = 0.11177 eV), pointing to its reactivity under electrophilic conditions, as shown in S5 Table. These distinct parameters illuminate the underlying electronic behavior of these ligands, highlighting a spectrum of reactivity profiles that could influence their utility in different chemical contents.

#### Molecular dynamic (MD) simulations, comprehensive analysis of structural flexibility and stability.

In this study, MD simulations and free energy calculations were utilized to investigate the binding modes and interaction mechanisms of the DNA gyrase B-DI31, tyrosyl-tRNA synthetase-DI31, and PBP4-DI22 complexes with differing efficacies. A detailed comparison, shown in Fig 8, highlights significant structural changes in compounds that substantially improve their binding affinity. In the DNA gyrase B-DI31 complex, two hydroxyl groups and one methyl-hydroxyl group attached to the tetrahydrofuran ring formed hydrogen bonds (Fig 8 B). In tyrosyl-tRNA synthetase-DI31, the imidazo [4,5-d] pyrazole group of DI31 forms hydrogen bonds with the amino group of H50 and the amino group of G193 (Fig 8 D). The hydroxyl groups attached to the tetrahydrofuran ring of DI31 formed hydrogen bonds with D40, D80 and the methyl-hydroxyl group formed H-bonds with D40 and Y170. The PBP4-DI22 complex binds tightly to active site residues (mainly S75, F241, Y291, and S262). The oxygen attached to the furan ring of DI22 forms two hydrogen bonds with S75 and N141. The cyclopentane ring forms a hydrogen bond with Y291, and the carboxylic group DI22 forms one hydrogen bond with the amino group of S262, as shown in (Fig 8 G). Docking studies have shown that these two inhibitors assume similar curve-shaped conformations within their respective binding pockets. Consequently, DI31 and DI22 inhibitors were selected for further MD simulations and binding free energy studies to determine the critical structural features required for selective DNA gyrase B, tyrosyl-tRNA synthetase, and PBP4 inhibition, thus laying the groundwork for developing more effective DNA gyrase B, tyrosyl-tRNA synthetase, and PBP4 inhibitors.

To evaluate the dynamic stability of our systems and verify the effectiveness of the sampling method, we monitored the root-mean-square deviation (RMSD) of the initial structures over 100 ns of the MD simulations. RMSD evaluations indicated that all systems, including the DNA gyrase B-DI31, tyrosyl-tRNA synthetase-DI31, and PBP4-DI22 complexes, reached equilibrium within the first 5 ns. Notably, the RMSD values for the Cα atoms of the protein DNA gyrase B, the backbone atoms of the binding pocket, and the heavy atoms of the ligand (DI31) stabilized at average values of approximately 2.5, 1.0, and 2.0 Å, respectively. These values are visually presented in (Fig 11 A), showcasing the stability of the systems, which forms a solid basis for further analyses such as hydrogen bonding free energy and energy decomposition based on conformations sampled from to 5–100 ns. The RMSD values for the Cα atoms of the tyrosyl-tRNA synthetase, the backbone atoms of the binding pocket, and the heavy atoms of the ligand (DI31) stabilized at average values of about 2.0 Å, 1.5 Å, and 1.5 Å, respectively, as shown in (Fig 13 C). In the case of DI22, the RMSD values for the Cα atoms of the protein (PBP4), the backbone atoms of the binding pocket, and ligand (DI22) stabilized at average values of about 2.0, 1.0, and 0.5 Å, respectively (Fig 11 E). The consistency of these conformations was further validated by overlaying the coordinates of representative MD-simulated snapshots onto their initial structures, as depicted in Fig 11 B (DNA gyrase B-DI31), Fig 11 D (tyrosyl-tRNA synthetase-DI31), and Fig 11 F (PBP4-DI22). This structural assessment confirmed that all complexes, including DNA gyrase B-DI31, tyrosyl-tRNA synthetase-DI31, and PBP4-DI22, maintained stability throughout the simulation, with all ligands retaining their initial conformation and essential hydrogen bonds with the active site region residues intact. These results affirm the precision of our MD simulation results in advancing binding free energy studies, providing valuable insights into the complex interaction mechanisms of these inhibitors with bacterial proteins.

**Fig 12:**
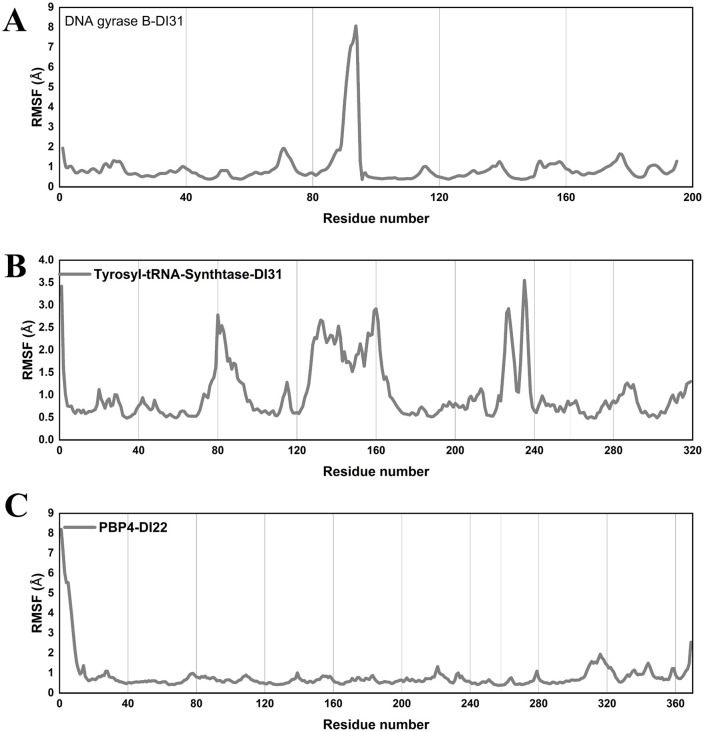
Root mean fluctuation (RMSF) curve for(A) DNA gyrase B-DI31, (B) Tyrsoyl-tRNA synthetase-DI31 (C) PBP4-DI22.

**Fig 13:**
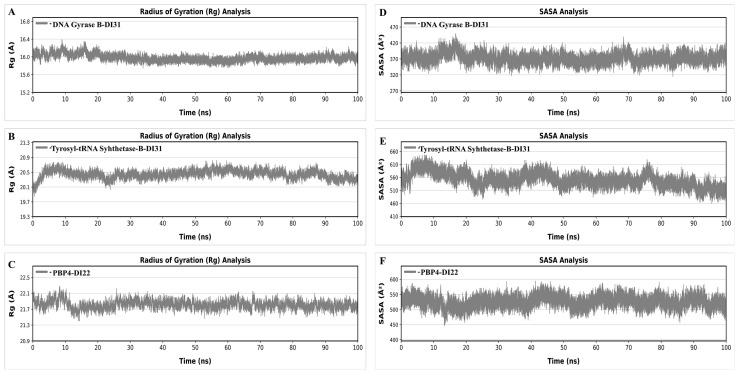
Plots for radius of gyration (ROG) of all complexes(A) DNA gyrase B-DI31, (B) Tyrsoyl-tRNA-synthetase-DI31 (C) PBP4-DI22. Solvent accessible surface area (SASA) for complexes. (D) DNA gyrase B-DI31, (E) Tyrsoyl-tRNA-synthetase-DI31 (F) PBP4-DI22.

The root-mean-square fluctuation (RMSF) analysis covering all complexes DNA gyrase B-DI31, tyrosyl-tRNA synthetase-DI31, and PBP4-DI22 interactions, as shown in Fig 12 A, 12B, and 12C, respectively, outlines the dynamic profiles and RMSF distribution across the protein structures of all evaluated systems. These dynamics exhibited consistent patterns, with areas within the DNA gyrase B residues N46, E50, D73, R76, K103, V120, and S121 showing pronounced fluctuations. Specifically, the active site residue K103 displayed higher fluctuations in both non-bonded and bonded interactions with DNA Gyrase B, whereas the active site residue, encompassing 45–50, 70–80, 116–120, 130–140 and 160–180 exhibited significant fluctuations in the system bonded with DI31. This observation suggests that ligand DI31 binding increases the mobility of residues 110–115, indicating lower fluctuation but enhanced flexibility between the active site regions in the presence of DNA Gyrase B, as shown in Fig 12 A. In tyrosyl-tRNA synthetase-DI31, residues D80, P81, K84, I131, Y140, E160, K231, and G238 showed higher fluctuations, whereas the active site residue encompassing 20–40, 75–83, 110–120, 130–160, 230–240 and 280–285 exhibited significant fluctuations in the system bonded with DI31. This observation suggests that ligand DI31 binding increases the mobility of residues 50–60, 90–100, 170–180, 260–270 and 290–310 indicating lower fluctuation but enhanced flexibility between the active site regions in the presence of tyrosyl-tRNA synthetase, as shown in Fig 12 B. Additionally, the active site residues D80 and G238 displayed higher fluctuations in both non-bonded and bonded interactions with tyrosyl-tRNA synthetase. In contrast, DI31 binds tightly to the residues present in the binding pocket between the α helical domain and the long variable arm of tyrosyl-tRNA synthetase and displayed stability across both tyrosyl-tRNA synthetase systems, emphasizing their crucial role in maintaining structural integrity upon ligand binding. Notably, the α-helical domain and long variable arm residues in receptor-ligand bonded systems exhibited a heightened fluctuation amplitude compared to those in the non-bonded tyrosyl-tRNA synthetase configuration, highlighting the significant dynamic influence of ligand binding on the mobility of this critical region. In PBP4-DI22, residues S75, E114, F241, S262, D264, and Y291 showed fluctuations, whereas the active site residue encompassing 70–80, 100–110, 150–160, 220–230, 260–280 and 310–320 exhibited significant fluctuations in the system bonded with DI22. This observation suggests that ligand DI22 binding increases the mobility of residues 40–50, 120–130, 190–210 and 285–290 indicating lower fluctuation but enhanced flexibility between the active site regions in the presence of PBP4. Additionally, the active site residues S75, E114, F241, R280, and K319 displayed higher fluctuations in both non-bonded and bonded interactions with PBP4, as shown in Fig 12 C. In contrast, DI22 binds tightly with the active site residue present between the N-terminus and N-terminal transpeptidase domain of PBP4 displayed stability, emphasizing their crucial role in maintaining structural integrity upon ligand binding.

To assess the influence of ligand binding on the structural stability of the protein, the radius of gyration (Rg) of DNA Gyrase B, tyrosyl-tRNA synthetase-DI31 and PBP4-DI22 were monitored during the simulation, as shown in Fig 13. The average Rg values calculated for DNA Gyrase B-DI31, tyrosyl-tRNA synthetase-DI31 and PBP4-DI22 were approximately 16.1 Å, 20.6 Å and 22.0 Å respectively, showing notable consistency. This stability suggests that the binding of these ligands does not cause significant alterations in the overall protein structure, such as unfolding or expansion. Moreover, the Rg values of DNA Gyrase B-DI31, tyrosyl-tRNA synthetase-DI31, and PBP4-DI22 remained within a tight range of 15.8 to 16.3 Å, 20.1 to 21.8 Å and 21.6 to 22.1 Å throughout the simulation, reinforcing the strong structural integrity of all proteins. These observations indicate that the interaction of complexes DNA Gyrase B, tyrosyl-tRNA synthetase-DI31, and PBP4-DI22 maintains the native conformation, which is vital for their inhibitory function by preserving the structure of the active site needed for effective ligand recognition. Additional understanding was obtained by analyzing the solvent-accessible surface area (SASA), providing deeper insights into the dynamics between DNA Gyrase B, tyrosyl-tRNA synthetase, and PBP4 with the investigated inhibitors, as illustrated in Fig 13. The SASA of DNA Gyrase B-DI31, tyrosyl-tRNA synthetase-DI31, and PBP4-DI22 were roughly 370 Å², 580 Å², and 540 Å², respectively, demonstrating a stable interaction within these complexes. The consistency in these measurements of all complexes highlights the effectiveness of DI31 and DI22 as potential inhibitors of DNA Gyrase B, tyrosyl-tRNA synthetase, and PBP4.

#### Binding free energy calculations by MMPBSA/MMGBSA and energy decomposition analysis.

**Fig 14:**
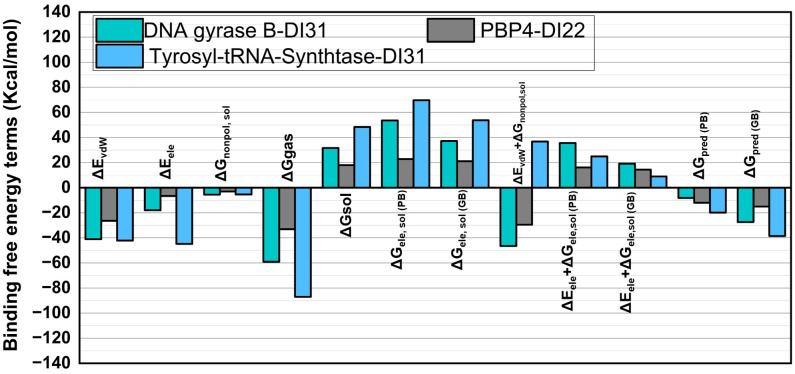
Binding free energy (Kcal/mol) terms of DNA gyrase B-DI31, Tyrsoyl-tRNA-synthetase-DI31 and PBP4-DI22.

Based on the system stability confirmed by the RMSD fluctuations shown in Fig 14, 10,000 snapshots were randomly extracted from the 1–100 ns interval of the MD simulation for the binding free energy calculations. The binding affinities of the selected compounds to DNA Gyrase B, tyrosyl-tRNA synthetase, and PBP4 were evaluated using both the MM/PBSA and MM/GBSA methods. The binding free energies calculated with MM/PBSA for DNA Gyrase B-DI31, tyrosyl-tRNA synthetase-DI31, and PBP4-DI22 complexes were −27.3659, −38.5752, and −15.0456 kcal/mol, respectively (Table 6). These results support the robustness of ΔG_pred_ (GB/PB) values obtained from MM/GB/PB/SA methods over those from traditional docking studies, confirming the validity of our computational approach in predicting the binding efficiency of potential DNA Gyrase B, tyrosyl-tRNA synthetase, and PBP4 inhibitors. The ability of the MMGB/PBSA method to decompose the total binding free energy into individual components provides valuable insights into ligand-receptor binding dynamics. As illustrated in Fig 14, the polar solvation energies (ΔE_ele_, _sol_) were positive, counterbalancing the favorable electrostatic energies (ΔE_ele_) observed in the gas phase across all complexes. This balance results in favorable combined electrostatic contributions (ΔG_ele_ + ΔG_ele_, _sol_) that support ligand-receptor complex formation. In contrast, the van der Waals interactions and nonpolar solvation energies (ΔE_vdW_ + ΔG_nonpol_, _sol_) contributed to negative values, enhancing the binding affinity of DI31. Notably, the ΔE_vdW_ values exceeded the ΔE_ele_ term in all systems, underscoring the importance of optimizing van der Waals and nonpolar interactions to improve the inhibitory potency of DI31 and DI22 inhibitors.

**Table 6:** Binding free energy (Kcal/mol) terms of DNA gyrase B-DI31, Tyrsoyl-tRNA-synthetase-DI31 and PBP4-DI22.

Complex	DNA Gyrase B-DI31	Tyrosyl-tRNA synthetase-DI31	PBP4-DI22
**Δ*E***_***vdW***_^***a***^	−41.0167	−42.1692	−26.4052
**Δ*E***_**ele**_^***a***^	−18.01	−44.7889	−6.653
**Δ*G***_**nonpol, sol**_^***a***^	−5.4989	−5.3852	−3.0518
**Δggas**	−59.0267	−86.9582	−33.0582
**ΔG**_**sol**_	31.6608	48.3829	18.0126
**Δ*G***_**ele, sol (PB)**_^***a***^	53.6435	69.7452	22.839
**Δ*G***_**ele, sol (GB)**_^***a***^	37.1597	53.7682	21.0644
**Δ*E***_***vdW***_ **+ Δ*G***_**nonpol,sol**_^***a***^	−46.5156	−47.5544	−29.457
**Δ*E***_**ele**_ **+ Δ*G***_**ele,sol (PB)**_^***a***^	35.6335	24.9563	16.186
**Δ*E***_**ele**_ **+ Δ*G***_**ele,sol (GB)**_^***a***^	19.1497	8.9793	14.4114
**Δ*G***_**pred (PB)**_^***b***^	−8.156	−19.8765	−12.0256
**Δ*G***_**pred (GB)**_^***b***^	−27.3659	−38.5752	−15.0456

The presence of several hydrophobic residues, such as R76, P79, I78 and K103 (DNA Gyrase B-DI31), A39, D40, G49, H50, P53, D80, K84, Y170, G193 and Q196 (tyrosyl-tRNA synthetase-DI31) and E114, L115, S116, A182, F241, S262, S263, D264 and Y291(PBP4-DI22) aligns with the observation that hydrophobic interactions play a crucial role in determining binding efficiency. While the electrostatic contributions are less dominant compared to the van der Waals and nonpolar solvation contributions, they still serve as a key factor in facilitating interactions within the complexes: DNA Gyrase B-DI31, tyrosyl-tRNA synthetase-DI31, and PBP4-DI22. Analysis of the DNA Gyrase B-DI31, tyrosyl-tRNA synthetase-DI31, and PBP4-DI22 complexes further exemplifies the predominance of van der Waals interactions in modulating inhibitory potency, with the ΔE_vdW_ term showing significant negative values for DNA Gyrase B-DI31 (−41.067 kcal/mol), tyrosyl-tRNA synthetase-DI31(−42.1692 kcal/mol), and PBP4-DI22 (−26.4052 kcal/mol) complexes. These findings highlight that although electrostatic interactions play a role in the binding process, van der Waals and hydrophobic interactions have a more significant impact on the inhibitory potential of DI31 and DI22 inhibitors. This analysis provides valuable insights into the binding mechanisms and suggests strategic approaches for designing more effective inhibitors of DNA Gyrase B, tyrosyl-tRNA synthetase, and PBP4 by prioritizing the enhancement of van der Waals and hydrophobic interactions.

Overall results and discussion summarized in S6 Table. It includes the DCM and MeOH extracts prepared from the plant material, with the DCM extract showing higher antibacterial activity in both ZOI and MIC assays. Subsequent *in silico* analysis identified DI10, DI31, and DI22 as lead compounds with strong binding to multiple bacterial targets, supported by ADMET, DFT, and molecular dynamics studies. These integrated findings suggest that the compounds present in the DCM extract are likely responsible for the observed antibacterial activity.

## Conclusion

*D. inermis* extracts (DCM and MeOH) were evaluated for antibacterial activity against *S. aureus*, *E. coli*, *P. aeruginosa*, *B. subtilis*, *S. typhi*, and *E. aerogenes*, with DCM extract showing superior potency. The pronounced efficacy of DCM underscores the richness of its bioactive constituents, as revealed by GC-MS (DCM extract) and HPLC (MeOH extract) analyses, identifying diverse secondary metabolites including alkaloids, cannabinoids, terpenoids, sesquiterpenoids, phenols, and flavonoids that likely act synergistically to inhibit bacterial growth [93]. Molecular docking demonstrated that compounds DI10 (alkaloid), DI22 (sesquiterpene), and DI31 (terpenoid) from DCM extract exhibited strong binding affinities to multiple bacterial targets, including DNA gyrase B, tyrosyl-tRNA synthetase, PBP2X, PBP4, and DHFR, highlighting their potential as effective antibacterial inhibitors. Structural analyses showed that these compounds form key hydrogen bonds and interactions for specificity and stability. ADMET predictions indicated favorable intestinal absorption, suggesting good systemic bioavailability. DFT and MESP analyses revealed significant electronic variations and dipole moments, supporting diverse binding potentials. MD simulations and binding free energy calculations further confirmed stable interactions of DI31 and DI22 with DNA gyrase B, tyrosyl-tRNA synthetase, and PBP4 via van der Waals and hydrophobic contacts. Collectively, these findings establish *D. inermis* as a promising natural source of antibacterial agents and provide a computational framework for optimizing its bioactive compounds toward developing novel therapeutics to combat microbial infections and antibiotic resistance.

## Supporting information

S1 Fig (A) Broth microdilution method (DCM extract of D. inermis at concentration (50-1.562 mg/ml) against gram positive and gram-negative bacteria). (B) Broth microdilution method (MeOH extract of *D. inermis* at concentration (50-1.562 mg/ml) against gram positive and gram-negative bacteria).
(TIF)

S2 Fig PLIP analysis of (A) DNA gyrase B-DI10, (B) DNA gyrase B-DI31, (C) Tyrosyl-tRNA synthetase-DI10, (D) Tyrosyl-tRNA synthetase-DI31, (E) PBP2X-DI10, (F) PBP2X-DI31, (G) PBP4-DI22, (H) PBP4-DI31, (I) DHFR-DI10, and (J) DHFR-DI31.
(TIF)

S1 Table One-way ANOVA Analysis of Antibacterial Activity of *D. inermis* Extracts Against Selected Bacterial Strains Compared with Gentamicin (10 µg), Showing Mean ± SD, F-values, p-values, and Significance. (DOCX)

S2 Table Antibacterial activity of *D. inermis* extracts compared by post-hoc comparisons. (DOCX)

S3 Table Identified drug-like compounds from *D. inermis* with their 2D structures. (DOCX)

S4 Table Docking scores of all docked compounds with DNA gyrase B, Tyrosyl-tRNA synthetase, PBP2X, PBP4, and DHFR. (DOCX)

S5 Table Parameters used for DFT analysis of the docked compounds. (DOCX)

S6 Table Summary of *D. inermis* extracts and lead compound analysis (*in vitro* and *in silico* studies). (DOCX)

## Abbreviations

MD: Molecular Dynamics
MMGBSA: Molecular Mechanics Generalized Born Surface Area
MMPBSA: Molecular Mechanics Poisson-Boltzmann Surface Area
BFE: Binding Free Energy
RMSD: Root Mean Square Deviation
RMSF: Root Mean Square Fluctuation
ADMET: Absorption, Distribution, Metabolism, Excretion, Toxicity.
