# Supplementary figures and images for: Identification of antimicrobial compounds in Dipsacus inermis via phytochemical profiling, in vitro assessment, and advanced computational techniques
Source: PLoS One. 2026 Feb 6;21(2):e0341424. doi: 10.1371/journal.pone.0341424 (PMC12880709; doi:10.1371/journal.pone.0341424)

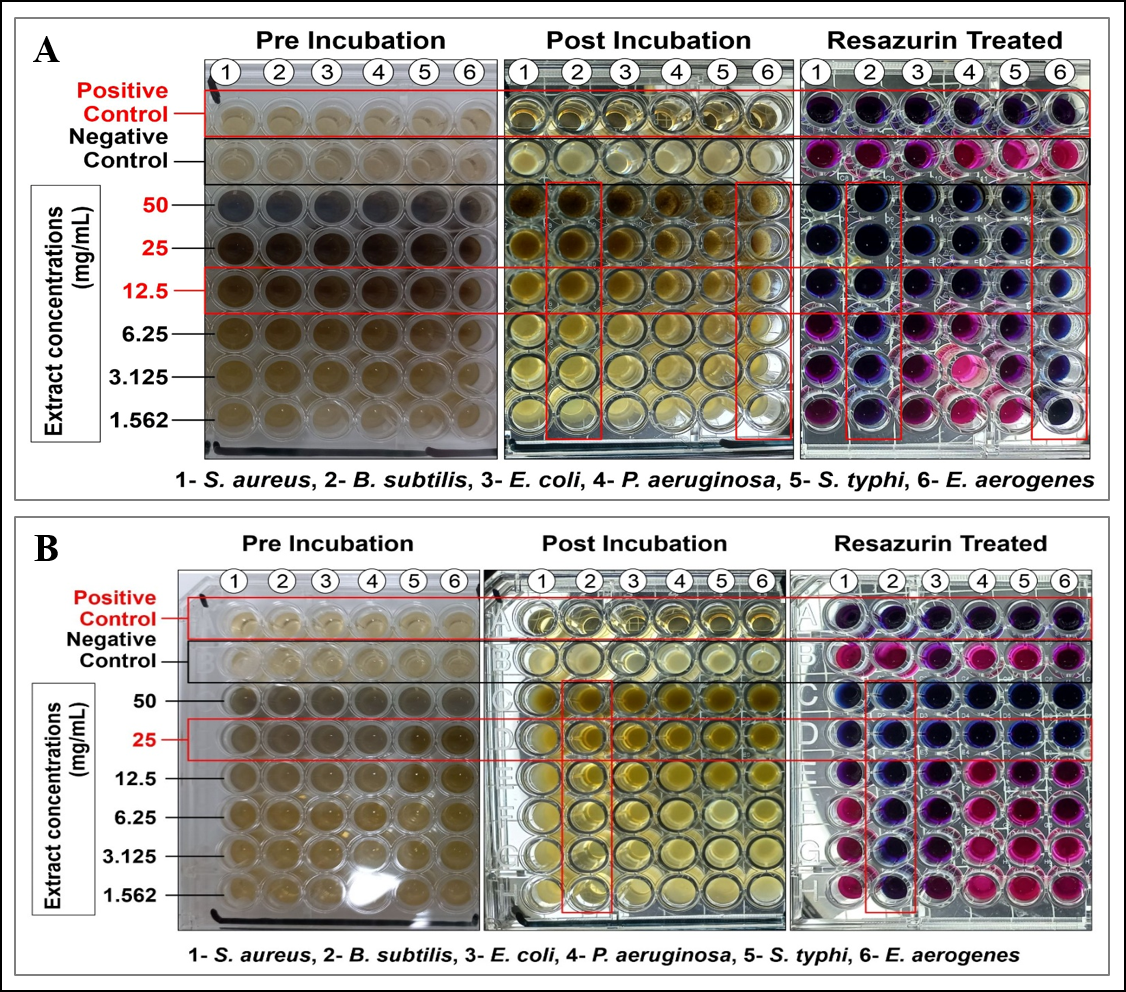

Supplement: S1 Fig — (A) Broth microdilution method (DCM extract of D. inermis at concentration (50-1.562 mg/ml) against gram positive and gram-negative bacteria). (B) Broth microdilution method (MeOH extract of D. inermis at concentration (50-1.562 mg/ml) against gram positive and gram-negative bacteria). (TIF) [file pone.0341424.s001.tif]

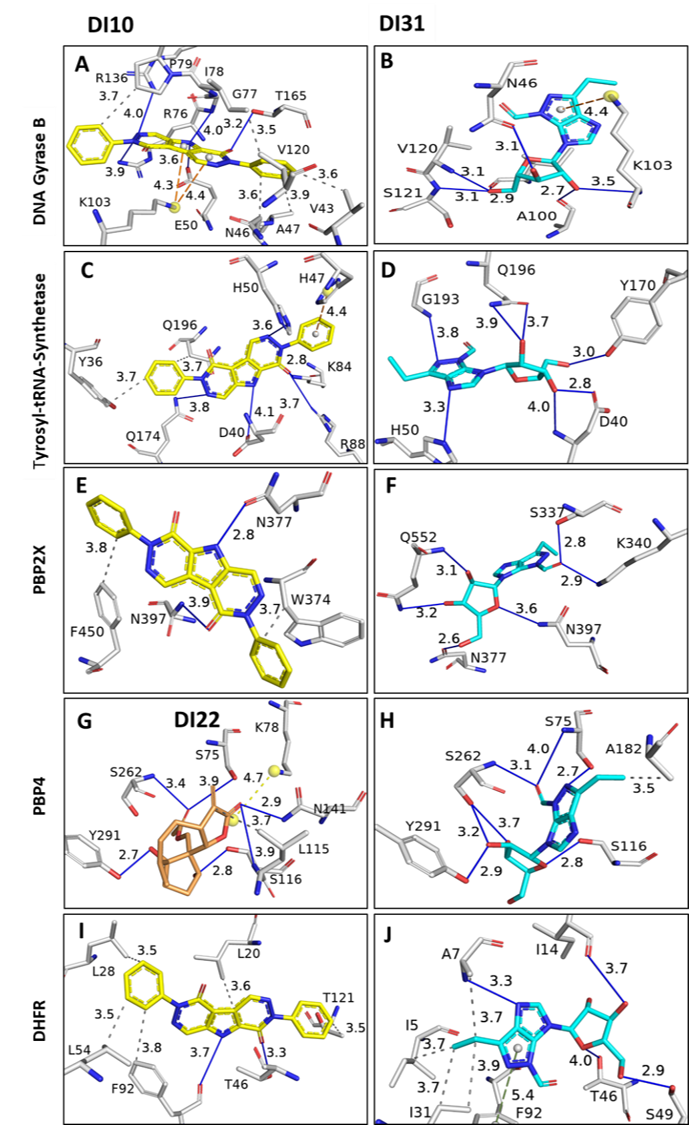

Supplement: S2 Fig — (A) DNA gyrase B-DI10, (B) DNA gyrase B-DI31, (C) Tyrosyl-tRNA synthetase-DI10, (D) Tyrosyl-tRNA synthetase-DI31, (E) PBP2X-DI10, (F) PBP2X-DI31, (G) PBP4-DI22, (H) PBP4-DI31, (I) DHFR-DI10, and (J) DHFR-DI31. (TIF) [file pone.0341424.s002.tif]
