# Supplementary material for: Identification of antimicrobial compounds in Dipsacus inermis via phytochemical profiling, in vitro assessment, and advanced computational techniques
Source: PLoS One. 2026 Feb 6;21(2):e0341424. doi: 10.1371/journal.pone.0341424 (PMC12880709; doi:10.1371/journal.pone.0341424)
Supplement: S1 Table — (DOCX) [file pone.0341424.s003.docx]

**S1 Table.** One-way ANOVA Analysis of Antibacterial Activity of D. inermis Extracts Against Selected Bacterial Strains Compared with Gentamicin (10 µg), Showing Mean ± SD, F-values, p-values, and Significance.

| **Bacteria** | **Extract (µg/mL)** | **Mean ± SD** | **F-value** | **p-value** | **Significance** |
| --- | --- | --- | --- | --- | --- |
| ***S. aureus*** | DCM 100 | 17.87 ± 0.23 | 194.65 | 0.000153 | *** |
|  | DCM 50 | 13.87 ± 0.23 | 18.83 | 0.01226 | * |
|  | MeOH 100 | 17.17 ± 0.29 | 54.54 | 0.00179 | ** |
|  | MeOH 50 | 12.13 ± 0.23 | 170.74 | 0.000198 | *** |
| ***E. coli*** | DCM 100 | 15.93 ± 0.11 | 93.81 | 0.000636 | *** |
|  | DCM 50 | 12.93 ± 0.11 | 876.53 | 0.00000775 | **** |
|  | MeOH 100 | 15.80 ± 0.34 | 20.57 | 0.01053 | * |
|  | MeOH 50 | 11.13 ± 0.23 | 7954.62 | 0.000000095 | **** |
| ***P. aeruginosa*** | DCM 100 | 16.83 ± 0.29 | 112.53 | 0.000447 | *** |
|  | DCM 50 | 13.10 ± 0.17 | 191.64 | 0.000158 | *** |
|  | MeOH 100 | 15.17 ± 0.29 | 4.94 | 0.0903 | ns |
|  | MeOH 50 | 9.07 ± 0.11 | 1720.72 | 0.00000202 | **** |
| ***B. subtilis*** | DCM 100 | 16.10 ± 0.17 | 165.97 | 0.000209 | *** |
|  | DCM 50 | 11.10 ± 0.17 | 1277.67 | 0.00000366 | **** |
|  | MeOH 100 | 14.07 ± 0.12 | 16.68 | 0.01504 | * |
|  | MeOH 50 | 8.93 ± 0.11 | 5677.97 | 0.000000186 | **** |
| ***S. typhi*** | DCM 100 | 16.93 ± 0.11 | 25.10 | 0.00744 | ** |
|  | DCM 50 | 13.87 ± 0.23 | 162.56 | 0.000218 | *** |
|  | MeOH 100 | 15.07 ± 0.10 | 553.47 | 0.0000194 | **** |
|  | MeOH 50 | 9.93 ± 0.11 | 10351.88 | 0.0000000559 | **** |
| ***E. aerogenes*** | DCM 100 | 17.87 ± 0.23 | 64.05 | 0.00132 | ** |
|  | DCM 50 | 13.83 ± 0.29 | 247.37 | 0.0000955 | *** |
|  | MeOH 100 | 14.10 ± 0.18 | 351.60 | 0.0000476 | *** |
|  | MeOH 50 | 10.60 ± 0.18 | 4365.79 | 0.000000314 | **** |

**Note:** * = p < 0.05, ** = p < 0.01, *** = p < 0.001, **** = p < 0.0001; ns = not significant.
