# Supplementary material for: Identification of antimicrobial compounds in Dipsacus inermis via phytochemical profiling, in vitro assessment, and advanced computational techniques
Source: PLoS One. 2026 Feb 6;21(2):e0341424. doi: 10.1371/journal.pone.0341424 (PMC12880709; doi:10.1371/journal.pone.0341424)
Supplement: S2 Table — (DOCX) [file pone.0341424.s004.docx]

**S2 Table:** **Antibacterial activity of *D. inermis* extracts compared by post-hoc comparisons**.

| **Bacterium** | **Pair** | **Mean ± SD** | **F-value** | **p-value** | **Significance** |
| --- | --- | --- | --- | --- | --- |
| ***S. aureus*** | DCM 100 vs MeOH 100 | 17.87 ± 0.23 vs 17.17 ± 0.29 | 6.97 | 0.037 | * |
| ***S. aureus*** | DCM 50 vs MeOH 50 | 13.87 ± 0.23 vs 12.13 ± 0.23 | 57.62 | 0.0007 | ** |
| ***S. aureus*** | DCM 100 vs DCM 50 | 17.87 ± 0.23 vs 13.87 ± 0.23 | 240.0 | 0.00002 | *** |
| ***S. aureus*** | MeOH 100 vs MeOH 50 | 17.17 ± 0.29 vs 12.13 ± 0.23 | 337.0 | 0.000005 | **** |
| ***E. coli*** | DCM 100 vs MeOH 100 | 15.93 ± 0.11 vs 15.80 ± 0.34 | 0.15 | 0.71 | ns |
| ***E. coli*** | DCM 50 vs MeOH 50 | 12.93 ± 0.11 vs 11.13 ± 0.23 | 82.5 | 0.0004 | ** |
| ***E. coli*** | DCM 100 vs DCM 50 | 15.93 ± 0.11 vs 12.93 ± 0.11 | 300.0 | 0.00002 | *** |
| ***E. coli*** | MeOH 100 vs MeOH 50 | 15.80 ± 0.34 vs 11.13 ± 0.23 | 210.0 | 0.000005 | **** |
| ***P. aeruginosa*** | DCM 100 vs MeOH 100 | 16.83 ± 0.29 vs 15.17 ± 0.29 | 32.8 | 0.002 | ** |
| ***P. aeruginosa*** | DCM 50 vs MeOH 50 | 13.10 ± 0.17 vs 9.07 ± 0.11 | 488.0 | 0.000001 | **** |
| ***P. aeruginosa*** | DCM 100 vs DCM 50 | 16.83 ± 0.29 vs 13.10 ± 0.17 | 181.0 | 0.00001 | *** |
| ***P. aeruginosa*** | MeOH 100 vs MeOH 50 | 15.17 ± 0.29 vs 9.07 ± 0.11 | 830.0 | 0.0000001 | **** |
| ***B. subtilis*** | DCM 100 vs MeOH 100 | 16.10 ± 0.17 vs 14.07 ± 0.12 | 56.5 | 0.0008 | ** |
| ***B. subtilis*** | DCM 50 vs MeOH 50 | 11.10 ± 0.17 vs 8.93 ± 0.11 | 112.0 | 0.0003 | ** |
| ***B. subtilis*** | DCM 100 vs DCM 50 | 16.10 ± 0.17 vs 11.10 ± 0.17 | 342.0 | 0.00001 | *** |
| ***B. subtilis*** | MeOH 100 vs MeOH 50 | 14.07 ± 0.12 vs 8.93 ± 0.11 | 568.0 | 0.000001 | **** |
| ***S. Typhi*** | DCM 100 vs MeOH 100 | 16.93 ± 0.11 vs 15.07 ± 0.10 | 61.2 | 0.0007 | ** |
| ***S. Typhi*** | DCM 50 vs MeOH 50 | 13.87 ± 0.23 vs 9.93 ± 0.11 | 211.0 | 0.00001 | *** |
| ***S. Typhi*** | DCM 100 vs DCM 50 | 16.93 ± 0.11 vs 13.87 ± 0.23 | 144.0 | 0.00002 | *** |
| ***S. Typhi*** | MeOH 100 vs MeOH 50 | 15.07 ± 0.10 vs 9.93 ± 0.11 | 810.0 | 0.0000001 | **** |
| ***E. aerogenes*** | DCM 100 vs MeOH 100 | 17.87 ± 0.23 vs 14.10 ± 0.18 | 118.0 | 0.00001 | *** |
| ***E. aerogenes*** | DCM 50 vs MeOH 50 | 13.83 ± 0.29 vs 10.60 ± 0.18 | 71.5 | 0.0003 | ** |
| ***E. aerogenes*** | DCM 100 vs DCM 50 | 17.87 ± 0.23 vs 13.83 ± 0.29 | 222.0 | 0.000005 | **** |
| ***E. aerogenes*** | MeOH 100 vs MeOH 50 | 14.10 ± 0.18 vs 10.60 ± 0.18 | 92.0 | 0.0001 | **** |

**Note:** * = p < 0.05, ** = p < 0.01, *** = p < 0.001, **** = p < 0.0001; ns = not significant.
