# Supplementary material for: Identification of antimicrobial compounds in Dipsacus inermis via phytochemical profiling, in vitro assessment, and advanced computational techniques
Source: PLoS One. 2026 Feb 6;21(2):e0341424. doi: 10.1371/journal.pone.0341424 (PMC12880709; doi:10.1371/journal.pone.0341424)
Supplement: S3 Table — (DOCX) [file pone.0341424.s005.docx]

**S3 Table. Identified drug like compounds from *D. inermis* with their 2D structures**

| **DI1** | 9-Octadecenoic acid, (2-phenyl-1,3-dioxolan-4-yl)methyl ester, cis- |  |
| --- | --- | --- |
| **DI2** | Linoleic acid ethyl ester |  |
| **DI3** | Acetic acid, 4-hydroxy-cyclohexyl ester |  |
| **DI4** | (25S)-3Beta-acetoxy-5alpha,22beta-spirost-9(11)-en-12beta-ol |  |
| **DI5** | 2-(1-Methyl-2-nitroethyl)cyclohexanone |  |
| **DI6** | 13-(Acetyloxy)tridecyl acetate |  |
| **DI7** | 8-Azabicyclo[3.2.1]octane, 8-acetyl- |  |
| **DI8** | Octadecanoic acid, 4-hydroxy-, methyl ester |  |
| **DI9** | 10àH-Ambros-11(13)-en-12-oic acid, 6á,15-dihydroxy-4-oxo-, ç-lactone, acetate |  |
| **DI10** | 2,7-Diphenyl-1,6-dioxopyridazino[4,5:2',3']pyrrolo[4',5'-d]pyridazine |  |
| **DI11** | 2,4-dinitro-N-[(E)-nonadecan-2-ylideneamino]aniline |  |
| **DI12** | Methyl 3-(acetyloxy)-20-hydroxyurs-12-en-28-oate |  |
| **DI13** | 3,4-dihydroxy-2-(3-methylbutanoyl)-5-(3-methylbut-2-enyl)-4-[(E)-4-methylpent-2-enoyl]cyclopent-2-en-1-one |  |
| **DI14** | Tricyclo[5.2.2.0(1,6)]undecan-3-ol, 2-methylene-6,8,8-trimethyl- |  |
| **DI15** | Caryophyllene oxide |  |
| **DI16** | 4-[(4-Aminophenyl)sulfonyl]-1,2-benzenediamine |  |
| **DI17** | Tetracyclo[6.3.2.0(2,5).0(1,8)]tridecan-9-ol |  |
| **DI18** |  |  |
| **DI19** | Isocolumbin |  |
| **DI20** | N-(4-Hydroxyphenyl)retinamide |  |
| **DI21** | 5H-Cyclopropa[3,4]benz[1,2-e]azulen- 5-one, 3,9,9a-tris(acetyloxy)-3-[(acetyloxy)methyl]-2-chloro-dodecahydro-4a,7b-dihydroxy-1,1,6, 8-tetramethyl-, |  |
| **DI22** | 10àH-Ambros-11(13)-en-12-oic acid, 1,4á,6á,15-tetrahydroxy-, ç-lactone, 15-acetate |  |
| **DI23** | 5H-Cyclopropa[3,4]benz[1,2-e]azulen-5 -one,2,9,9a-tris(acetyloxy)-3-[(acetyloxy)methyl] -dode Cahydro -3,4a,7b-trihydroxy-1,1,6,8-tetramethyl- |  |
| **DI24** | 1- Oxaspiro[4.4]non-8-ene-4,7-dione, 9-hydroxy-2-isopropyl-8-isovaleryl-6-(3-methyl-2-butenyl)- |  |
| **DI25** | 3,4-dihydroxy-5-(3-methylbut-2-en-1-yl)-2-(3-methylbutanoyl)-4-(4-methylpent-3-enoyl)cyclopent-2-en-1-one |  |
| **DI26** | 4,5,10-trihydroxy-4a-(hydroxymethyl)-2,2,6a,6b,9,9,12a-heptamethyl-icosahydropicen-3-yl (Z)-2-methylbut-2-enoate |  |
| **DI27** | Digitoxin |  |
| **DI28** | 7,8,12-Tri-O-acetyl ingo |  |
| **DI29** | 1-(1-Hydroxy-3,3-dimethyl-2-[(1E)-3-methyl-1,3-butadienyl]cyclopentyl)ethanone |  |
| **DI30** | Menthol, 1'-(butyn-3-one-1-yl)-, (1R,2S,5R)- |  |
| **DI31** | Pyrazole[4,5-b]imidazole, 1-formyl-3-ethyl-6-á-d-ribofuranosyl- |  |
| **DI32** | Picrotoxinin |  |
| **DI33** | Estra-1,3,5(10)-trien-17á-ol |  |
| **DI34** | 3a,6b,8a-trihydroxy-2a-(hydroxymethyl)-1,1,5,7-tetramethyl-4-oxo-dodecahydro-1H cyclopropa[5',6']benzo [1',2':7,8]azuleno[5,6-b]oxiren-8-yl acetate |  |
| **DI35** | 6-Amino-4-(3,4-dichlorophenyl)-3-propyl-1,4-dihydropyrano[2,3-c]pyrazole-5-carbonitrile |  |
| **DI36** | Flunarizine |  |
| **DI37** | 6H-Dibenzo[b,d]pyran-1-ol, 6a,7,8,10a-tetrahydro-6,6,9-trimethyl-3-propyl-, (6aR-trans)- |  |
| **DI38** | 1,3-Benzenediol, 2-[3-methyl-6-(1-methylethenyl)-2-cyclohexen-1-yl]-5-pentyl-, (1R-trans)- |  |
| **DI39** | Dronabinol |  |
| **DI40** | D-Homo-24-nor-17-oxachola-20,22-dien  e-3,16-dione, 7-(acetyloxy) -triepoxy-4 ,4,8-trimethyl-, |  |
| **DI41** | Safranin |  |
| **DI42** | Corticosterone |  |
| **DI43** | dihydroxy-7-(hydroxymethyl)- tetramethyl-15-oxo-4-tetracyclo-pentadeca-2,7-dienyl] dodecanoate |  |
| **DI44** | dihydroxy-3-(hydroxymethyl)-1,1,6,8-tetramethyl-5-oxo-decahydro-9aH-cyclopropa[3,4]benzo[1,2-e]azulen-9a-yl isobutyrate |  |
| **DI45** | 14á-Pregn-5-ene-pentol, 11-acetate 12-isovalerate |  |
| **DI46** | 3-(acetoxymethyl)-5,7b-dihydroxy-1,1,6,8-tetramethyl-decahydro-9aH-cyclopropa[3,4]benzo[1,2-e]azulene-9,9a-diyl diacetate |  |
| **DI47** | 3- Hydroxyspirost-8-en-11-one |  |
| **DI48** | 3a,6b,8a-trihydroxy-2a-(hydroxymethyl)-1,1,5,7-tetramethyl-4-oxo-dodecahydro-1H cyclopropa[5',6']benzo[1',2':7,8]  azuleno[5,6-b]oxiren-8-yl acetate |  |
| **DI49** | 2-(2-(2-methoxy-1,1-dimethyl-6-oxooctahydro-2H-2,4a-(epoxymethano)naphthalen-5-yl)ethyl)-2-methyl-3-oxocyclopentyl acetate |  |
| **DI50** | ethyl 4-(3,7,12-trihydroxy-10,13-dimethylhexadecahydro-1H-cyclopenta[a]phenanthren-17-yl)pentanoate |  |
| **DIH1** | Berberine |  |
| **DIH2** | Ferulic acid |  |
| **DIH3** | Reserpine |  |
| **DIH4** | Caffiec acid |  |
| **DIH5** | Rutin |  |
| **DIH6** | Chlorogenic acid |  |
