# Supplementary material for: Identification of antimicrobial compounds in Dipsacus inermis via phytochemical profiling, in vitro assessment, and advanced computational techniques
Source: PLoS One. 2026 Feb 6;21(2):e0341424. doi: 10.1371/journal.pone.0341424 (PMC12880709; doi:10.1371/journal.pone.0341424)
Supplement: S4 Table — (DOCX) [file pone.0341424.s006.docx]

**S4 Table. Docking scores of all docked compounds with DNA gyrase B, tyrosyl-tRNA synthetase, PBP2X, PBP4 and DHFR.**

| **Compounds** | **DNA Gyrase B** | **Tyrosyl-tRNA-Synthetase** | **PBP2X** | **PBP4** | **DHFR** | **Compounds** | **DNA Gyrase B** | **Tyrosyl-tRNA-Synthetase** | **PBP2X** | **PBP4** | **DHFR** |
| --- | --- | --- | --- | --- | --- | --- | --- | --- | --- | --- | --- |
| **DOCKING SCORES (Kcal/mol)** | | | | | | | | | | | |
| **DI1** | -4.471 | -4.954 | -5.278 | -4.044 | -5.415 | **DI26** | -2.565 | -3.951 | -4.616 | -3.564 | -2.136 |
| **DI2** | -5.347 | -2.671 | -3.823 | -2.934 | -3.486 | **DI27** | -4.238 | -1.864 | -8.326 | -2.145 | -1.967 |
| **DI3** | -5.494 | -6.496 | -4.79 | -5.075 | -5.647 | **DI28** | -1.045 | -1.937 | -3.606 | -2.451 | 2.438 |
| **DI4** | -1.676 | -4.678 | -4.285 | -2.81 | -5.569 | **DI29** | -5.76 | -5.188 | -5.492 | -5.362 | -6.24 |
| **DI5** | -4.606 | -5.114 | -4.409 | -5.277 | -5.222 | **DI30** | -6.411 | -5.178 | -4.072 | -5.926 | -5.864 |
| **DI6** | -3.826 | -2.715 | -3.218 | -3.329 | -4.319 | **DI31** | -8.573 | -6.051 | -6.387 | -6.631 | -7.704 |
| **DI7** | -4.684 | -5.414 | -5.291 | -5.947 | -6.554 | **DI32** | -1.546 | -5.767 | -6.303 | -2.632 | -6.369 |
| **DI8** | -3.398 | -3.86 | -4.632 | -3.312 | -4.741 | **DI33** | -6.955 | -6.083 | -5.27 | -6.506 | -6.97 |
| **DI9** | -2.345 | -4.644 | -4.831 | -4.989 | -3.403 | **DI34** | -3.648 | -5.357 | -6.916 | -5.064 | -5.427 |
| **DI10** | -6.603 | -6.633 | -6.041 | -5.556 | -7.866 | **DI35** | -4.999 | -7.723 | -4.715 | -5.668 | -5.211 |
| **DI11** | -3.942 | -4.53 | -4.575 | -3.945 | -4.552 | **DI36** | -5.354 | -5.916 | -4.407 | -4.987 | -6.652 |
| **DI12** | -1.765 | -3.966 | -3.14 | -2.902 | -2.843 | **DI37** | -6.691 | -5.848 | -4.826 | -5.926 | -6.745 |
| **DI13** | -6.14 | -5.163 | -6.062 | -6.24 | -6.838 | **DI38** | -6.241 | -5.955 | -4.629 | -4.024 | -5.763 |
| **DI14** | -3.246 | -5.091 | -5.017 | -4.007 | -6.128 | **DI39** | -6.508 | -5.666 | -4.448 | -5.92 | -6.368 |
| **DI15** | -4.266 | -5.028 | -4.257 | -4.464 | -5.625 | **DI40** | -2.634 | -2.841 | -5.078 | -2.713 | -3.043 |
| **DI16** | -6.246 | -5.482 | -5.715 | -4.133 | -6.426 | **DI41** | -7.562 | -5.355 | -5.543 | -4.537 | -7.327 |
| **DI17** | -3.621 | -5.596 | -4.838 | -5.041 | -6.725 | **DI42** | -5.475 | -3.922 | -5.023 | -4.554 | -5.046 |
| **DI18** | -5.269 | -6.22 | -5.475 | -4.606 | -6.89 | **DI43** | -4.395 | -4.59 | -4.666 | -2.955 | -5.779 |
| **DI19** | -4.015 | -3.105 | -4.798 | -3.838 | -4.203 | **DI44** | -3.458 | -4.603 | -3.646 | -4.355 | -4.864 |
| **DI20** | -4.779 | -6.832 | -5.842 | -5.513 | -5.646 | **DI45** | -3.293 | -5.607 | -4.29 | -3.758 | -6.768 |
| **DI21** | -2.386 | -3.794 | -4.922 | -3.882 | -3.318 | **DI46** | -3.496 | -4.85 | -4.29 | -4.701 | -4.164 |
| **DI22** | -1.825 | -6.034 | -4.626 | -6.625 | -2.552 | **DI47** | -2.432 | -4.389 | -4.91 | -4.131 | -3.298 |
| **DI23** | -2.493 | -3.953 | -4.671 | -3.059 | -2.143 | **DI48** | -1.643 | -1.472 | -2.301 | -2.546 | -2.152 |
| **DI24** | -3.284 | -3.843 | -5.847 | -5.393 | -6.199 | **DI49** | -2.432 | -4.438 | -4.994 | -3.97 | -5.031 |
| **DI25** | -3.467 | -4.544 | -5.914 | -5.57 | -7.439 | **DI50** | -2.654 | -4.3 | -5.515 | -4.191 | -5.339 |
| **DIH1** | -2.665 | -3.876 | -4.876 | -3457 | -2.765 | **DIH4** | -2.293 | -4.607 | -4.29 | -3.758 | -2.768 |
| **DIH2** | -1.765 | -2.765 | -3.876 | -4.876 | -3.65 | **DIH5** | -1.293 | -3.607 | -3.39 | -2.758 | -3.768 |
| **DIH3** | -3.655 | -2.876 | -3.87 | -1.45 | -4.98 | **DIH6** | -0.045 | -1.737 | -2.606 | -3.451 | 1.438 |
