# Supplementary material for: Identification of antimicrobial compounds in Dipsacus inermis via phytochemical profiling, in vitro assessment, and advanced computational techniques
Source: PLoS One. 2026 Feb 6;21(2):e0341424. doi: 10.1371/journal.pone.0341424 (PMC12880709; doi:10.1371/journal.pone.0341424)
Supplement: S5 Table — (DOCX) [file pone.0341424.s007.docx]

**S5 Table. Parameters used for DFT analysis of the docked compounds.**

| **Parameters for DFT analysis** | **Ligands** | | |
| --- | --- | --- | --- |
|  | **DI10** | **DI22** | **DI31** |
| **Dipole moment (Debye)** | 4.284 | 10.432 | 4.21 |
| **HOMO (eV)** | -0.2235 | -0.24546 | -0.24054 |
| **LUMO (eV)** | -0.05571 | -0.05966 | -0.05075 |
| **Energy gap (eV)** | 0.16779 | 0.18594 | 0.18979 |
| **Ionization (eV)** | 0.2235 | 0.24546 | 0.24054 |
| **Electron affinity (eV)** | 0.05571 | 0.05966 | 0.05075 |
| **Electronegativity (χ) (eV)** | 0.13961 | 0.15256 | 0.14565 |
| **Electrochemical potential μ (eV)** | -0.13961 | -0.15256 | -0.14565 |
| **Hardness η (eV)** | 0.08389 | 0.0929 | 0.0949 |
| **Softness σ** | 11.91997 | 10.76482 | 10.53678 |
| **Electrophilicity** | 0.11602 | 0.12536 | 0.11177 |
