# Supplementary material for: Identification of antimicrobial compounds in Dipsacus inermis via phytochemical profiling, in vitro assessment, and advanced computational techniques
Source: PLoS One. 2026 Feb 6;21(2):e0341424. doi: 10.1371/journal.pone.0341424 (PMC12880709; doi:10.1371/journal.pone.0341424)
Supplement: S6 Table — (DOCX) [file pone.0341424.s008.docx]

**S6 Table. Summary of *D. inermis* extracts and lead compound analysis (*in vitro* and *in silico* studies).**

| **Stage** | **Observation / Finding** | **Lead Compounds / Key Values** |
| --- | --- | --- |
| **Plant Extraction** | Two extracts prepared: DCM (325 mg), MeOH (410 mg) | - |
| **In Vitro Antibacterial Activity** | Assessed by ZOI (Gentamicin 20 µg) and MIC (Vancomycin 10 µg); ANOVA applied; DCM more active | ZOI (mm): DCM 18–22, MeOH 12–15 MIC (µg/mL): DCM 25–50, MeOH 50–100 DCM showed largest inhibition & lowest MIC |
| **In Silico Screening (Docking)** | GC-MS compounds docked against DNA Gyrase B, Tyrosyl-tRNA Synthetase, PBP2X, PBP4, DHFR; multi-target analysis | DI10, DI31 (multi-target), DI22 (PBP4) Docking scores (kcal/mol): DI10 -9.8 to -10.5, DI31 -9.5 to -10.2, DI22 -8.6 DCM compounds dominate high binding energies |
| **ADMET Analysis** | Evaluated pharmacokinetics, toxicity, drug-likeness for all GC-MS compounds; focus on leads | DI10, DI31, DI22; favorable absorption, non-toxic, good drug-likeness |
| **DFT & MESP Studies** | Electronic properties and reactive sites analyzed; rationalizes ligand-protein interactions | DI10, DI31, DI22; HOMO-LUMO gaps: 4.2–4.5 eV MESP shows nucleophilic/electrophilic sites match protein binding pockets |
| **Molecular Dynamics Simulation** | Stability and binding behavior of protein-ligand complexes | DI10, DI31, DI22  RMSD: 2.0–3.0 Å  RMSF: residues 1–150 flexible  Rg: 18.5–19.2 Å (compact)  H-bonds: 3–5 persistent  MM-PBSA binding free energy: -45 to -52 kcal/mol |
| **Overall Insight** | Workflow: extracts → antibacterial assay → in silico identification → stability & interaction confirmed | DCM extracts contain DI10, DI31, DI22; likely responsible for antibacterial activity; integrated approach validates leads |
